# Multi‐Objective Catalyst Discovery in High‐Entropy Alloy Composition Space: The Role of Noble Metals on the Pareto Front for Oxygen Reduction Reaction

**DOI:** 10.1002/anie.8695284

**Published:** 2026-05-05

**Authors:** Mads K. Plenge, Ahmad Tirmidzi, Christian M. Clausen, Matthias Arenz, Jan Rossmeisl

**Affiliations:** ^1^ Center for High‐Entropy Alloy Catalysis (CHEAC) Department of Chemistry University of Copenhagen København Ø Denmark; ^2^ Department of Chemistry Biochemistry and Pharmaceutical Sciences University of Bern Bern Switzerland

**Keywords:** electrochemistry, heterogeneous catalysis, high‐entropy alloys, multiobjective optimization, oxygen reduction reaction

## Abstract

Discovering new materials for electrocatalytic energy conversion reactions is a key step toward energy sustainability. However, for catalysts to be viable in practice, they must perform in multiple, potentially conflicting objectives. We demonstrate this challenge for the acidic oxygen reduction reaction (ORR), where activity, stability, and material cost must be balanced. Using the continuous composition space of high‐entropy alloys (HEAs) together with our established models for activity and dissolution, we identify a Pareto‐optimal set of ORR catalysts within the Ag─Au─Cu─Ir─Pd─Pt─Rh─Ru system via multiobjective Bayesian optimization. Additionally, we introduce a fine‐tuned machine learning model that predicts adsorption energies for alloys spanning 12 elements and 9 adsorbates. Our results show that alloying expands the hypervolume spanned by the Pareto front, consisting of low‐ to medium‐entropy alloys composed primarily of Ag, Au, Cu, Pd, and Pt. We further propose an approach for analyzing the Pareto front by quantifying the loss in hypervolume when critical elements (Au, Pd, and Pt) are removed, clarifying their relative contributions to optimal performance. This work highlights the need to consider all relevant objectives in catalyst optimization and the advantage of HEAs as a powerful platform for multiobjective catalyst discovery.

## Graphical abstract

Pareto optimal compositions of alloy catalyst for oxygen reduction reaction are uncovered through multi‐objective Bayesian optimization of activity, stability, and material cost in an eight‐element high‐entropy alloy composition space. The substantial Pareto front obtained is compared to experimental literature and analyzed to elucidate the roles and importance of elements.

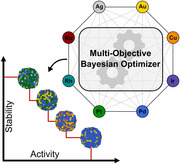

## 1 Introduction

New catalyst materials are a key aspect toward advancing the utilization of renewable energy [1, 2, 3]. An important and highly studied reaction is the cathodic oxygen reduction reaction (ORR) in hydrogen fuel cells, for which major challenges persist, one of which is designing catalysts that remain active throughout many catalytic cycles [4, 5]. For the ORR in proton‐exchange membrane fuel cells, the operative potential window coupled with the acidic electrolyte provides a harsh electrochemical environment resulting in high catalyst degradation, for which dissolution is a major degradation mechanism [6, 7, 8]. A related challenge is reducing the amount of Pt, the benchmark catalyst, whose cost hinders large scale applications [9, 10]. Thus, the challenge for the acidic ORR is to design catalysts that are active, stable, and cost‐efficient versus Pt. However, these individual objectives may not be reconcilable, and therefore, one must consider Pareto optima, optimal trade‐offs between conflicting objectives.

High‐entropy alloys (HEAs), multielemental alloys of five or more metals in near equimolar ratios, have gained increasing interest as catalysts [11, 12]. A key property of HEAs is their compositional tuneability ascribing HEAs as a “Catalyst discovery platform” [13]. Despite the high complexity of HEAs, great advances have been achieved in understanding their tunable catalytic activity arising from adsorption energy distributions [14, 15], which can be predicted through machine learning [13, 16]. Moreover, Bayesian optimization has been shown both theoretically and experimentally as an effective approach to discover new catalysts utilizing the vast HEA composition space [17]. Through this work, we argue that only considering a single objective, e.g., catalytic activity, neglects an important strength of HEAs, namely providing a large selection of Pareto optima, which we first displayed for catalytic activity versus element scarcity in the quinary Ag─Ir─Pd─Pt─Ru HEA system [18]. Pareto fronts can be uncovered through multi‐objective optimization, which has gained increasing interest within materials science, including HEAs, as material properties are often conflicting [19]. Within HEA catalysis, multiobjective Bayesian optimization (MOBO) of compositions have been demonstrated to efficiently uncover Pareto fronts of HEA systems: In experimental work, Jenewein et al. utilized MOBO to uncover Pareto optimal compositions with respect to activity and stability of Co─Mn─Sb─Sn─Ti oxide for acidic oxygen evolution reaction (OER) [20], while theoretical work used MOBO for HEA catalysts for ORR, employing our framework for activity [16] with alloy cost and mixing‐entropy as additional objectives [21]. More recently, Zhang et al. demonstrated an evolutionary approach to uncover HEA activity‐stability Pareto front for OER while highlighting the need for electrochemically relevant stability metrics in theoretical approaches [22]. Thus, significant potential remains in including modelling of stability to uncover activity‐stability‐cost Pareto fronts for ORR relevant for application. Although stability during electrochemical operation is a vital objective for a catalyst's application, a lack of understanding persists concerning the effect of the electrochemical environment and the resulting catalyst degradation of HEAs [23]. We recently addressed this by proposing a framework for simulating dissolution of alloy particles, including HEAs [24]. With this new model at hand, together with our model for activity utilizing a large, fine‐tuned adsorption regression model, and the molar cost of the alloys, we combine these objectives in a MOBO framework to estimate the Pareto front of the Ag─Au─Cu─Ir─Pd─Pt─Rh─Ru HEA system for ORR. The quality of the set of Pareto optimal solutions can be quantified by the n‐dimensional volume spanned by the Pareto front relative to a reference point. This quality indicator was first suggested by Zitzler and Thiele and today commonly referred to as the hypervolume (HV) [25, 26]. Here we will utilize the HV both as the maximization objective in MOBO and in comparing Pareto fronts across HEA spaces. Thereby, we investigate the impact of excluding Pt and Pd for activity, and Au for stability on the Pareto front to illustrate their importance, and perhaps necessity, despite the conventional goal of minimizing their usage.

## 2 Results and Discussion

### 2.1 Adsorption Regression by Fine‐Tuning

**FIGURE 1:**
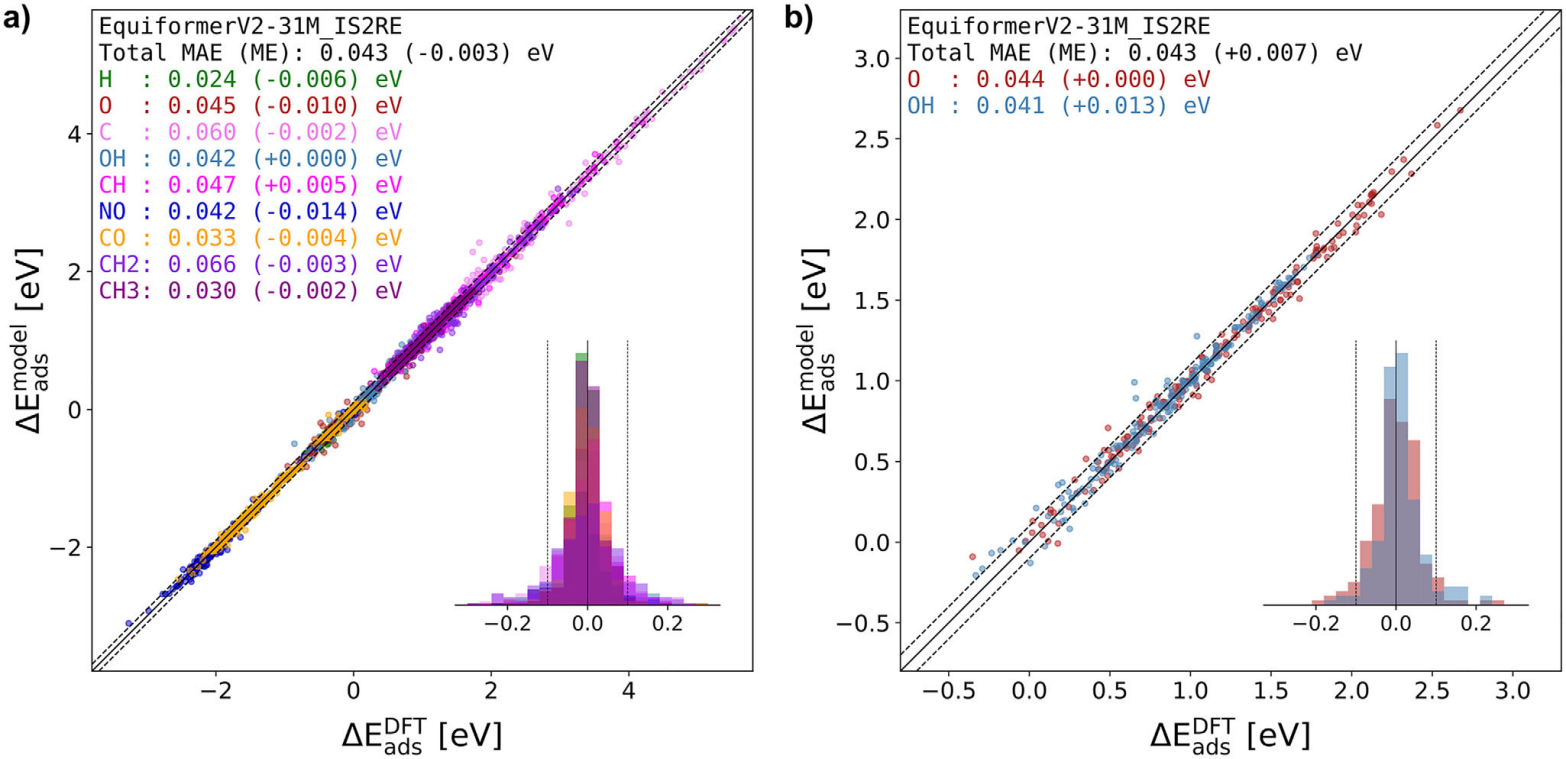
Parity plots for the HEA fine‐tuned of EquiformerV2 with 31 million parameters with a) showing the full 12‐element HEA system with nine adsorbates and b) showing the parity plot on the octonary test set containing only *O and *OH.

The adsorption regression is obtained from fine‐tuning a pretrained Open Catalyst Project [27] model (EquiformerV2 [28]) to an extensive 12‐element HEA dataset containingnine adsorbates. The adsorption datasets and fine‐tuning are described in the computational methods in the Supporting Information. The fine‐tuned model is obtained by the parameters yielding the lowest validation mean absolute error (MAE), which occurs at epoch 148 with an MAE of 0.041 eV. The corresponding learning curve is shown in Figure S1 in the Supporting Information. The performance of the model on the HEA and the octonary test sets is shown as parity plots in Figure 1. Additional parity plots for the lower entropy datasets are provided in Figure S2 in the Supporting Information. For the HEA test set, Figure 1a, a total MAE of 0.043 eV across all adsorbates on the test set is obtained. MAEs of 0.039 and 0.042 for the binary and ternary test sets demonstrate that the accuracy is retained for lower entropy alloys. Moreover, an even lower MAE of 0.012 eV is obtained for the pure metal test set. In this work, we employ this model for *O and *OH adsorption energies with Ag─Au─Cu─Ir─Pd─Pt─Rh─Ru alloy compositions. As illustrated by Figure 1b, the accuracy for *O and *OH are preserved within this compositional subsystem with MAEs of 0.044 and 0.041 eV, respectively.

Thus, this fine‐tuned adsorption energy model provides substantial flexibility by including nine adsorbates in the 12‐element composition space with high accuracy against DFT across the 12‐element composition space and its subspaces. Hence it can be utilized for a wide range of problems.

### 2.2 The Pareto Front of Pristine Metals

**FIGURE 2:**
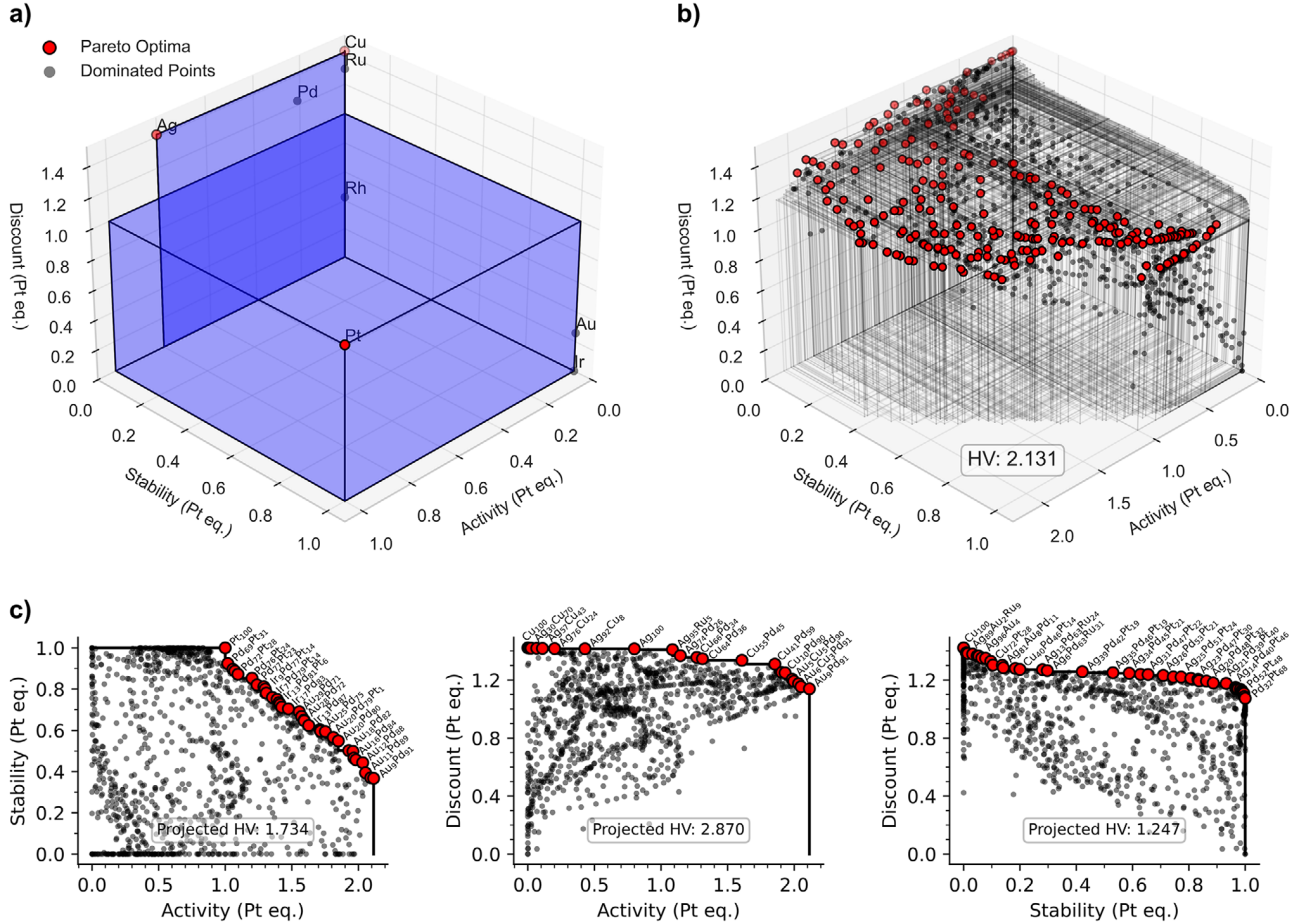
Pareto fronts of Ag─Au─Cu─Ir─Pd─Pt─Rh─Ru catalysts for ORR as given by the objectives normalized to Pt with a) showing the single metal catalysts Pareto front, b) the alloy Pareto front obtained from all rounds of MOBO utilizing the HEA composition space, and c) the projection of b) into two‐dimensional Pareto fronts with annotated compositions in atomic percent (at. %). In a) and b) the dominated space of each Pareto optima is illustrated with boxes to provide a sense of space. Where Pareto optima are close in c), the displayed labels are representative selections.

To assess the potential benefits of alloying, the results should be compared to the Pareto front of the pristine metals shown in Figure 2a. The activity and stability are obtained through previously published models [16, 24], while the discount refers to the saving in cost relative to the most expensive element, Ir, in $/mol. The objective functions are described in computational methods in the Supporting Information. The objective values are normalized by the ones of Pt denoted as Pt equivalences (Pt eq.). The rationale for using Pt as the standard benchmark for ORR becomes clear from this comparison: Pt is the only element that simultaneously exhibits reasonable activity and stability. Consequently, it is the only contributor to the hypervolume (HV), referring to the (hyper)volume spanned by the Pareto front, which equals 1 under the chosen normalization relative to the origin. We consistently employ the origin as the reference point throughout this work as 0 is the minimum value for all objectives.

### 2.3 The Pareto Front of Ag─Au─Cu─Ir─Pd─Pt─Rh─Ru

Because of the vastness of the HEA compositions space, we employ MOBO to estimate the Pareto fronts of the full space and seven subspaces by maximizing the HV. See the computational methods in the Supporting Information for the methodology. In Figure 2b, the resulting Pareto front from all the collected samples with MOBO is shown, containing a total of 1094 unique samples. An HV of 2.131 versus Pt is obtained; a significant improvement by alloying versus the pristine metals. This is an as‐sampled HV, which contains noise on the objectives. Figure S3 in the Supporting Information displays the variability on the total HV due to the estimated noise showing negligible influence with a standard deviation of 0.01 by computing multiple HVs by adding Gaussian noise, at the inferred noise level of the Gaussian Processes (GPs), to the means of the posteriors acting as proxy for the true noiseless values. The small impact of noise on the HV value follows from small noises on the simulated objectives inferred to be 0.02 and 0.03 in Pt eq. units for activity and stability, respectively. The noise on the simulated objectives is a statistical uncertainty from sampling a finite HEA surface and number of nanoparticles, and, although small, may still influence which compositions are labeled as Pareto optimal. A straightforward methodology to reduce the influence of noise, where objective functions are more uncertain, could be to use the posterior means of the GPs to filter noise, which here results in a HV of 2.125. However, the homoscedastic noise level overestimates noise in cases, e.g., the pure metals, where the variance of the simulated objectives are close to zero or zero. We will therefore use the objective values as sampled in displaying the Pareto fronts and computing the HV.

To improve the visual interpretability of the Pareto front, the three‐dimensional Pareto front is projected to show the pairwise combination of objectives in Figure 2c with a representative selection of the Pareto optimal compositions annotated. However, in showing the Pareto fronts in objective pairs, it is important to note that many Pareto optimal compositions become hidden as the nuance of a third objective is lost, which emphasizes the need to consider all relevant objectives. The projected Pareto fronts obtained from individual MOBO runs are given in Figure S4 and the HV per iteration is given in Figure S5 in the Supporting Information. It is notable that the single metal Pareto optimal catalysts are still found to be Pareto optimal within this HEA space. Moreover, the front consists primarily of compositions containing Ag, Au, Cu, Pd, and Pt, suggesting the Ag─Au─Cu─Pd─Pt HEA composition space to be of specific interest for the ORR to balance activity, operational stability, and material cost. In fact, by only considering these elements, an HV of 2.120 is obtained spanning ∼99.5% of the total HV. Moreover, the Au─Pd─Pt ternary space alone covers 96% with a HV of 2.047. Their projected Pareto fronts containing all samples are shown in Figure S6 in the Supporting Information.

Focusing on activity versus stability, as Pt is deemed fully stable against dissolution by the model at these conditions, the front cannot be expanded in this objective direction. Meanwhile, in the activity direction the front is expanded to reach activities more than twice as active as Pt. The most stable Pareto optimal alloys that are more active (and more cost effective) than Pt, are Pd─Pt alloys, which are also known in literature to be able to provide comparable or enhanced activity versus Pt [29, 30, 31, 32, 33, 34, 35, 36, 37, 38]. Perhaps surprisingly, the Pareto optimal activity improvement is driven by alloys with ≥69 at.% Pd, rather than by alloying Pt. This observation highlights two effects: (1) Alloying a Pt surface to increase the activity of Pt through the ligand effect is ineffective due to the dilution of active sites. (2) As we recently reported [39], the disruption of the pristine Pd surface, favoring *O in the face‐centered cubic (fcc) hollow site, with weak *O‐binding elements facilitates on‐top *OH on Pd, which has near‐optimal binding energy, is a more effective approach than tuning the binding energy with ligands. It also suggests that, at least in the case of ORR, it is more advantageous to stabilize a highly active but unstable catalyst through alloying than vice versa. However, with the high amount of Pd, the electrochemical stability is expected to decrease drastically. Nevertheless, the composition with best activity is found to be Au_9_Pd_91_, which agrees with recent experimental findings showing Pd‐rich Au─Pd binary alloy as the optimum in the Ag─Au─Pd composition space for the alkaline ORR [40]. Moreover, the Pareto front suggests that Au is additionally advantageous for its ability to maintain stability with increasing Pd versus Pt. Au─Pd has also been shown to be great ORR catalysts elsewhere [41, 42, 43, 44].

By including the cost objective, unsurprisingly, Cu and Ag, the cheapest of the metals considered, become influential in alloys with predominantly Pd and Pt, whereas the activity‐stability front is dominated by Au, Pd, and Pt as well as a small area including Ir. Ag can provide the same effect on activity as Au in alloying with Pd [39] and Ag_10_Pd_90_ have been shown to exhibit high ORR activity in alkaline conditions relative to Pd [45]. However, it suffers from a stability of zero within this model in the applied acidic conditions and is therefore not prioritized in the MOBO sampling due to an individual HV of 0. Instead, the sampling has prioritized adding Pt, providing stability and thereby a nonzero HV. Other notable Pareto optimal alloys include Cu─Pd alloys. These alloys are not stable in acidic ORR conditions but may be more favorably applied in alkaline ORR where Cu is less prone to dissolve, which is supported by experimental results showing better catalytic performance in activity and stability compared to acidic ORR [46, 47, 48]. Moreover, Pd─Cu alloys have been reported to exhibit higher ORR activity than Pt/C in alkaline medium [36, 46, 47, 49], as predicted here.

### 2.4 The Pareto Fronts of Subspaces

**FIGURE 3:**
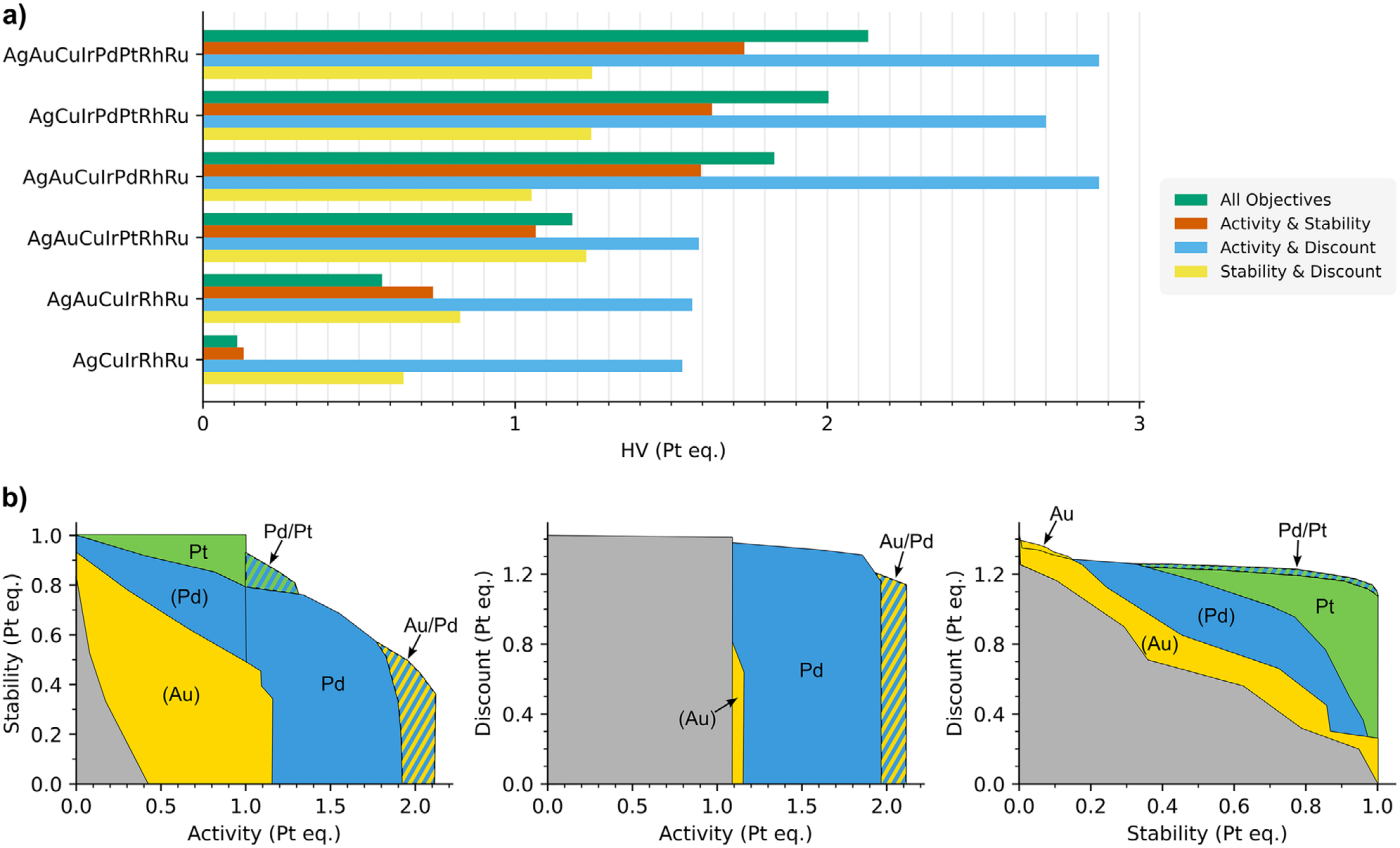
a) Comparisons of HVs between the octonary composition space and five selected subspaces. b) The projected Pareto fronts showing areas lost as a consequence of removing the labelled element. Elements in parentheses signify that the degradation dependents on removing critical elements in outer areas meanwhile shared areas suggest that removal of either element is critical. The innermost (grey) area reflects the HV of Ag─Cu─Ir─Rh─Ru.

From the Pareto front of the octonary alloy in Figure 2, Au, Pd, and Pt are the main components constituting the Pareto optimal alloys. By comparing HVs without these elements present, we obtain new insight into their relative importance given by their impact on the HV, and thus, on the quality of the Pareto front. To make this comparison, we estimated the HV of five subsystems using MOBO: Ag─Cu─Ir─Pd─Pt─Rh─Ru (no Au), Ag─Au─Cu─Ir─Pt─Rh─Ru (no Pd), Ag─Au─Cu─Ir─Pd─Rh─Ru (no Pt), Ag─Au─Cu─Ir─Rh─Ru (no Pd and Pt), and Ag─Cu─Ir─Rh─Ru (no Au, Pd, and Pt). The HVs are compared in Figure 3a, showing both the three‐objective HV and the two‐objective pairings. It should be noted that the HVs should only be compared within the same set of objectives.

The projected Pareto fronts of the subsystems containing all data within their respective composition space are given in Figure S7 and shown overlaid with the full space Pareto front in Figure S8. From these figures we have qualitatively drawn the HV‐areas lost by the removal of Au, Pd, and Pt in Figure 3b. The comparisons show that Pd has a much larger impact on the three‐objective HV than Au and Pt. This is unsurprising as Pd is in most of the found Pareto optimal compositions as seen in Figure 2c. However, most of the loss in HV arises from the loss in activity in both the activity‐stability and activity‐discount objective subspaces, making it evident that Pd is necessary to reach high activities relative to Pt. In fact, it seems most critical in the activity‐discount objective space where it accounts for almost half of the HV. In comparison, Au alone is not as critical to the overall HV. However, without the presence of Pd and Pt, it becomes vital for preserving the HV.

Although Au and Pt have similar degradational impact on the activity‐stability HV, their individual roles are reflected by their critical areas in Figure 3b. Au is critical to reach activities 2× that of Pt while preserving some stability, whereas Pt is critical to preserving moderate activity at high stability against dissolution. Moreover, Pt is the most vital element to the stability‐discount HV.

Removing both Pd and Pt reduces the total HV to less than that of pristine Pt with a negative impact larger than their individual sum. Thus, unsurprisingly, Pd and Pt are highly important for providing viable options of ORR catalysts. Even without Pd and Pt, the activity‐stability HV is retained close to that of Pt. Examining the Pareto front (Figure S7d) shows that the activity‐stability front consists of an Au─Cu gradient. In acidic conditions, retaining activity poses a challenge as it requires stabilizing Cu from dissolution. Nonetheless, we find their predicted competing activities with Pt without incorporating Pd and Pt intriguing, although they may be more suited for alkaline ORR, for which they have been demonstrated as active catalysts with ordered phases showing catalytic performance similar to the benchmark Pt [50, 51, 52, 53, 54].

Finally, having neither Au, Pd, nor Pt present leaves a detrimental impact on the total HV leaving only an HV of 0.110, meaning that 95% of the HV is lost. Thus, illustrating their necessity for a practical acidic ORR catalyst, at least within the scope of the applied models, despite the general goal to minimize their usage. We believe that analyzing the HV degradation from the included components, as done here, provides additional insight into their individual roles and may favorably be applied in similar multiobjective studies, including experimental studies. Moreover, it illustrates the importance of including the “right” elements in applications of multiobjective optimization of alloy catalysts. Solving this by including as many metals as possible may not be a favorable solution as it combinatorically increases the search space while these results suggest that most elements may not be necessary to include at all.

### 2.5 Pareto Optimal Alloy Composition for Oxygen Reduction Reaction

In Table S6 in the Supporting Information, we list all the 226 compositions estimated to be Pareto optimal within the Ag─Au─Cu─Ir─Pd─Pt─Rh─Ru composition space grouped into 19 clusters by a Gaussian mixture model. Notably, there are no HEAs found to be Pareto optimal, but instead low‐ to medium‐entropy alloys with 14 being quaternaries, 130 ternaries, and 79 binaries, as well as the three single metals Ag, Cu, and Pt. This highlights the versatility of the HEA composition space as a discovery platform but also suggests that HEAs by themselves may not be the most suitable alloys for the ORR even when considering multiple objectives. We recently discussed this tendency of HEAs not being the optimal compositions in activity within a composition space suggesting that the dilution of active sites limits the intrinsic catalytic activity [55]. Moreover, entropy is also not found to be a stabilizing factor in the dissolution simulation [24]. However, the fact that the majority of Pareto optimal compositions are ternary alloys suggest that the number of elements within the Pareto optimal compositions may increase with objectives as different objectives favor different metals.

In Table 1, the Pareto optimal clusters in Table S6 are summarized with literature references to experimental works. Notably, most of these systems have been experimentally studied for the ORR, which is in part due to the high prevalence of binary systems but also a testament to comprehensive literature for the ORR. Moreover, it validates the presented multiobjective framework and suggests that theoretical models can be utilized to guide experimental efforts toward regions of interest in the vast composition space. The literature references exclude studies solely focused on core‐shell particles, which is a common strategy for activity and stability enhancement for the ORR and thus represents a large fraction of the literature on ORR metal catalysts. Moreover, the dissolution of alloy nanoparticles can significantly change the surface composition, leading to core‐shell particles, impacting the activity through both ligand‐ and strain effects besides the loss in active surface area [7, 24, 56]. Including core‐shell catalysts in future work would therefore provide a more comprehensive multiobjective catalyst search but would require more complex modeling and an expanded dataset [57]. Likewise, the simulated objectives, in addition to being models, do not account for all variable aspects of the ORR such as the choice of electrolyte and potential variations. Moreover, the stability parameter, describing stability against dissolution, accounts for only one degradational pathway either through dissolution to the electrolyte or through Ostwald ripening. Although dissolution mediated degradation is a main mechanism necessary to model the degradation, e.g. shown for Pt/C [8], nanoparticle catalysts may also degrade through nondissolution driven mechanisms such as agglomeration and coalescence or particle detachment from the carbon support [58, 59, 60], for which modelling would require consideration of the stability of the support and its interactions with the catalyst material [61, 62]. Moreover, these mechanisms are also directly related to synthesis and electrode preparation, and the activity is affected by evolving surface composition, surface structure, and surface area due to the different degradation mechanisms. Thus, the stability parameter should be understood as a structure–property prediction that represents intrinsic electrochemical stability of nanoparticles with a defined composition, size, and structure under the given reaction conditions.

**TABLE 1:** Overview of Pareto optimal clusters listing the number of compositions in each cluster (#), the median and range of objective values relative to Pt, and experimental literature references to the alloy systems in various nanostructures for both acidic and alkaline ORR. The cluster names refer to the main elements in the compositions constituting the cluster (See Table S6 for the individual compositions). Bold indicates the location of the pure metal and parenthesis refers to elements present in most, but not all, compositions in the cluster.

		Median (min–max range) in Pt eq.			
Cluster	#	Activity	Stability	Discount	Refs.
Au─Pd	15	1.93 (1.56–2.11)	0.50 (0.37–0.69)	1.06 (0.95–1.14)	[40, 41, 42, 43, 44]
Ag─Cu─Pd(─Pt)	9	1.16 (0.94–1.41)	0.46 (0.02–0.63)	1.24 (1.21–1.31)	[64]
Pd─Ru	9	1.40 (1.02–1.93)	0.32 (0.23–0.36)	1.25 (1.18–1.27)	[65]
Ir─Pd─Ru	3	1.60 (1.60–1.74)	0.50 (0.41–0.61)	1.17 (1.08–1.19)	
Pd─Pt	28	0.93 (0.36–1.66)	0.93 (0.33–1.00)	1.15 (1.07–1.21)	[29, 30, 31, 32, 33, 34, 35, 36, 37, 38]
**Ag**(─Au)	6	0.58 (0.48–0.80)	0.05 (0.00–0.10)	1.36 (1.33–1.41)	[66, 67, 68]
Ag─Pd─Pt	41	0.94 (0.60–1.37)	0.77 (0.37–0.99)	1.20 (1.13–1.26)	[69]
Ir─Pd─Rh	4	1.84 (1.83–1.86)	0.47 (0.41–0.50)	1.13 (1.13–1.14)	
Cu─Pd─Pt	13	1.41 (1.11–1.58)	0.22 (0.08–0.46)	1.25 (1.21–1.29)	[70, 71]
**Pt**(─Ag)	13	0.69 (0.31–1.00)	1.00 (0.96–1.00)	1.01 (1.00–1.09)	[72, 73, 74, 75]
Au─Cu─Pd	7	2.00 (1.64–2.05)	0.28 (0.10–0.34)	1.17 (1.15–1.24)	[76]
Ir─Pd─Pt	22	1.51 (0.85–1.82)	0.67 (0.34–0.94)	1.11 (1.04–1.19)	[77]
Ag─Cu	6	0.09 (0.01–0.43)	0.00 (0.00–0.00)	1.42 (1.42–1.42)	[78, 79, 80, 81]
Ag─Ru	4	0.87 (0.72–1.09)	0.04 (0.00–0.09)	1.37 (1.32–1.41)	
Ir─Pd	7	1.77 (1.47–1.85)	0.56 (0.42–0.70)	1.11 (1.02–1.16)	[82, 83, 84, 85]
**Cu**(─Pt)	4	0.81 (0.00–0.85)	0.10 (0.00–0.14)	1.31 (1.28–1.42)	[86, 87, 88]
Cu─Pd	14	1.82 (1.27–1.93)	0.02 (0.00–0.18)	1.29 (1.19–1.36)	[36, 46, 47, 48, 49]
Ag─Au─Pd	11	0.97 (0.67–1.35)	0.06 (0.00–0.14)	1.34 (1.29–1.39)	[40]
Au─Pd─Pt	10	1.49 (0.65–1.85)	0.64 (0.33–0.99)	1.17 (1.12–1.19)	[89, 90]

Throughout this study, Pd‐rich Au─Pd alloys emerge as particularly promising candidates, as they uniquely span the most active region of the HV while remaining more cost‐efficient than Pt with the applied price data. The primary source of dissolution is attributed to low‐coordinated Pd‐atoms; consequently, alloy stability may be enhanced without compromising activity by selectively stabilizing these sites, for example through galvanic replacement of non‐facet Pd with Au to form a protective frame [24]. Alternatively, Pd─Au alloy nanoparticles can be straightforwardly synthesized and screened using electrodeposition approaches [63]. We view this as an exciting experimental challenge that holds great potential for ORR. Another highlighted metal combination worth further experimental study, although not listed in Table 1, is Au─Cu as, despite obvious dissolution stability challenges and heavy reliance on Au, they offer a Pd‐ and Pt‐free alternative.

### 2.6 Outlook on Multiobjective Optimization of Alloy Catalysts

The presented Pareto fronts are obtained through simulation‐based models, which cannot take all variables of experiments into account. However, they do demonstrate that the alloy compositions with the highest intrinsic activity are likely not the most stable against dissolution or the most cost efficient. Furthermore, they present invaluable guidelines for experimental studies. The optimal catalyst on the Pareto front may depend on the application. Therefore, experimental efforts in catalyst discovery should employ multi‐objective approaches. At the same time, it is important to recognize that not all objectives carry equal practical weight; for instance, expanding the hypervolume toward extreme stability at very low activity may not be valuable. In such cases, the hypervolume can be restricted or evaluated only above a relevant minimum activity threshold by changing the reference point.

From an experimental standpoint, the most important requirement when performing comparative multi‐objective optimizations is to follow standardized accelerated stress tests (ASTs) to evaluate catalyst degradation. Catalyst degradation can arise from various processes depending on the applied operating conditions [75, 91, 92, 93]. When metal dissolution is the primary degradation pathway—as in this computational study—potential step protocols between 0.6 and 0.95 or 1.0 V_RHE_ with holding times of 3 s are commonly used in both half‐cell and MEA testing [94]. However, in scanning flow cell setups coupled with inductively coupled plasma mass spectrometry (ICP‐MS), the upper potential limit is often increased. This is because metal dissolution is mainly triggered by repeated oxidation–reduction cycles, conditions that are typically avoided in industrial fuel‐cell applications such as automotive systems [95, 96, 97, 98].

Due to differences between the nature of experimental data and simulation‐based datasets, sampling strategies must be adapted to the higher noise levels, nonuniform data distributions, and practical constraints inherent to experimental conditions and elaborate experimental efforts to collect sufficient data points. These considerations also motivate hybrid strategies, where simulation‐based models are incorporated into experimental MOBO runs to guide exploration toward promising regions before committing to expensive measurements.

**FIGURE 4:**
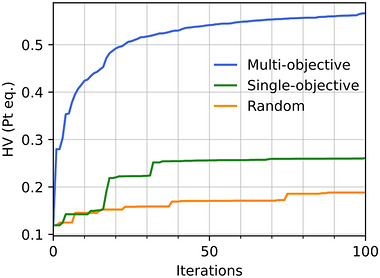
HV per iteration for different sampling strategies: multi‐objective, single‐objective (optimizing activity), and random sampling from a uniform Dirichlet distribution in the Ag─Au─Cu─Ir─Rh─Ru composition space.

The effectiveness of sampling by considering multiple objectives versus a single objective and random sampling in uncovering Pareto optima is illustrated in Figure 4. The figure shows the obtained HV after each iteration of sampling within the Ag─Au─Cu─Ir─Rh─Ru for 100 iterations. We use this space as example as the objectives within the space are more conflicting than, e.g., the full octonary space. For all three cases the same 26 initial samples are used: The six pure metals and 20 random compositions. The multiobjective case utilizes the described methodology for MOBO. The single‐objective is Bayesian optimization also utilizing the same methodology as MOBO but uses a single‐objective acquisition function to optimize for the activity only. The random sampling draws compositions from the uniform Dirichlet distribution and therefore considers no objectives. In summary, the figure demonstrates the effectiveness in improving the HV by MOBO and exposes the limitation of only optimizing a single objective when other objectives are also relevant. This consideration becomes even more critical experimentally, where each iteration carries substantial cost, making it essential to extract as much information as possible from every measurement.

## 3 Conclusion

We have presented a large adsorption energy dataset of density functional theory calculated structure relaxations of nine adsorbates on 12‐element HEA fcc (111) slabs, which was utilized to fine‐tune the pretrained EquiformerV2 (31 M) for the initial structure to relaxed energy task. The fine‐tuned adsorption energy model displays high accuracy with MAE of 0.043 eV across all adsorbates on the high‐entropy test set. Moreover, accuracy is preserved in the low‐entropy domain and within subdomains, making the model flexible by being applicable for a wide array of problems. Here we utilize it to predict adsorption energies of large extended alloy surfaces of *OH and *O to predict ORR activity for compositions within the Ag─Au─Cu─Ir─Pd─Pt─Rh─Ru composition space.

Through the combination of simulation models for electrochemical activity and stability of HEAs together with molar cost of the alloys, we have constructed a novel theoretical Pareto front for ORR within the Ag─Au─Cu─Ir─Pd─Pt─Rh─Ru composition space. The Pareto front demonstrates that alloying provides additional optimal tradeoffs between the objectives i.e., Pareto optima, compared to the pristine metals, for which Ag, Cu, and Pt remain Pareto optimal. The Pareto front contains primarily binary and ternary alloy systems already known and studied in literature, most notably including Au─Pd, Pd─Cu, and Pd─Pt. Moreover, we find that Ag─Au─Cu─Pd─Pt spans ∼99.5% of the hypervolume and Au─Pd─Pt spans ∼96% by themselves, suggesting great care should be taken in choosing relevant elements.

We investigate the importance of selected noble metals, Au, Pd, and Pt, by uncovering Pareto fronts from compositions spaces for which they are excluded. We find that excluding all three has a detrimental effect on the quality of the Pareto front expressed through its spanned hypervolume, demonstrating their combined importance. Moreover, by analyzing the degradational effect on the hypervolume and Pareto front from their exclusion, we provide a novel methodology for insight into their importance for each objective and role in forming the Pareto front. Au has the role of a stabilizing agent, especially important for stabilizing highly active Pd‐rich alloys, which are found to be the most active catalysts within the employed activity model. Pd and Pt are especially important for optimal activity versus stability trade‐offs: Pd‐alloys provide the most active catalyst but are deemed less stable against dissolution, whereas Pt‐alloys provide a more stability favored tradeoff. These properties are also reflected by their importance with combination of the cost related objective, where Pd is vital for activity‐cost tradeoffs while Pt is crucial for the stability‐cost tradeoffs.

Although none of the found Pareto optima are HEAs, the HEA composition space provides a design space for optimization to discover Pareto optimal alloy catalysts. Moreover, the resulting expanded Pareto front featuring a multitude of Pareto optimal compositions reveals a new strength of HEA catalysis as a multi‐objective discovery platform.

## Author Contributions

**Mads K. Plenge**: Conceptualization, methodology, writing – original draft, writing – review and editing, investigation, software, visualization, formal analysis, data curation. **Ahmad Tirmidzi**: writing – review and editing, validation, conceptualization. **Christian M. Clausen**: Writing – review and editing, methodology, software, data curation. **Matthias Arenz**: Writing – review and editing, supervision, funding acquisition, conceptualization. **Jan Rossmeisl**: Writing – review and editing, supervision, funding acquisition, project administration, resources, conceptualization.

## Conflicts of Interest

The authors declare no conflicts of interest.

## Supporting information




**Supporting File**: The authors have cited additional references within the Supporting Information [99–107].

## Data Availability

The full database for the 12‐element adsorption energy structure relaxations can be accessed here at doi.org/10.17894/ucph.e610ad78‐041a‐43e8‐8efb‐33f103702fce. Code and data for full reproduction can be accessed here: doi.org/10.17894/ucph.4dba68f1‐09a0‐40d5‐ad44‐9f3641e62318.
