# Supplementary material for: Multi‐Objective Catalyst Discovery in High‐Entropy Alloy Composition Space: The Role of Noble Metals on the Pareto Front for Oxygen Reduction Reaction
Source: Angew Chem Int Ed Engl. 2026 May 5;65(26):e8695284. doi: 10.1002/anie.8695284 (PMC13285469; doi:10.1002/anie.8695284)
Supplement: Supplementary file 1 — Supporting File: The authors have cited additional references within the Supporting Information [99–107]. [file ANIE-65-e8695284-s001.pdf]

Supporting Information

Multi-Objective Catalyst Discovery in High-Entropy Alloy Composition Space: The Role of Noble Metals on the Pareto Front for Oxygen Reduction Reaction

Mads K. Plenge[1], Ahmad Tirmidzi[2], Christian M. Clausen[1], Matthias Arenz[2], and Jan Rossmeisl[1,*]

[1]Center for High-Entropy Alloy Catalysis (CHEAC), Department of Chemistry, University of Copenhagen, 2100 Copenhagen, Denmark
[2]Department of Chemistry, Biochemistry and Pharmaceutical Sciences, University of Bern, 3012 Bern, Switzerland
[*]Corresponding author: jan.rossmeisl@chem.ku.dk

Contents

S1 Computational Methods **S3**
S1.1 Adsorption Energy Datasets S3
S1.2 Fine-tuning of Open Catalyst Project Adsorption Model S5
S1.3 Objectives S7
S1.4 Multi-Objective Bayesian Optimization S8

S2 Results **S9**
S2.1 Adsorption Energy Fine-tuning S9
S2.2 Multi-Objective Bayesian Optimization S11

References **S22**

List of Figures

S1 Learning curve . . . S9
S2 Parity plots of low-entropy test sets . . . S10
S3 Distribution of HVs from added noise . . . S11
S4 Pareto fronts of individual MOBO runs . . . S12
S5 HV per iteration of MOBO . . . S14
S6 Ag-Au-Cu-Pd-Pt and Au-Pd-Pt projected Pareto front . . . S14
S7 Pareto fronts of spaces with removed Au, Pd, and Pt . . . S15
S8 Pareto front comparison . . . S16

List of Tables

S1 Adsorbate site and gas reference . . . S3
S2 DFT specs . . . S4
S3 Adsorption energy datasets . . . S4
S4 Finetuning configurations . . . S5
S5 Metal prices . . . S7
S6 Pareto optimal compositions and their objective values . . . S17

S1 Computational Methods

S1.1 Adsorption Energy Datasets

As part of this work, we introduce an extensive HEA adsorption energy dataset, which we have utilized to fine-tune the EquiformerV2[1] model pretrained on the Open Catalyst Project (OCP) OC20 dataset[2] into an initial-structure-to-relaxed-energy (IS2RE) model following the methodology in Clausen et al.[3] The main dataset includes 9 adsorbates (*H, *O, *C, *OH, *CH, *NO, *CO, *CH2, and *CH3) on compositions within the 12-metal Ag-Au-Co-Cu-Ir-Ni-Os-Pd-Pt-Re-Rh-Ru HEA compositional system. In addition to the main HEA dataset, single metal, binary and ternary datasets were also constructed to function as test sets in the low-entropy regime. The dataset can be accessed here: `doi.org/10.17894/ucph.e610ad78-041a-43e8-8efb-33f103702fce`. Finally, an octonary test set for the utilized HEA system in this work, namely Ag-Au-Cu-Ir-Pd-Pt-Rh-Ru, with adsorbates *O and *OH were constructed to test the model performance within the applied application.

The adsorption energies were calculated from adsorbates placed on $3 \times 3 \times 5$ fcc(111) slabs with lateral periodic boundary conditions and a 10 Å vacuum layer above and below the slab constructed in the Atomic Simulation Environment[4]. The two bottom atomic layers have fixed positions. The lateral dimensions of the unit cell are scaled to the weighted mean of the lattice constants of the elements in the surface layer[5]. The database also contains the slabs without the adsorbates as well as molecular gas references of H_2, H_2O, CO, NO, and CH_4. The adsorption energy is calculated using equation S1 where $E_{slab+adsorbate}$ and E_{slab} are the relaxed total energies of the slab with and without the adsorbate, and E_{gas} is the gas phase reference calculated from the formulas listed in Table S1.

$$\Delta E_{ads} = E_{slab+adsorbate} - E_{slab} - E_{gas} \tag{S1}$$

A total of 2000 unique slabs were constructed for the main HEA dataset: 1000 with compositions drawn from a uniform Dirichlet distribution, 500 from a Dirichlet distribution with α-parameter of 0.2, and 500 with α-parameter of 0.1. Each adsorbate were placed twice on each of the slabs that converged (1920 slabs) in their respective sites (Table S1). The structure relaxations for the 9-adsorbate datasets were carried out in GPAW[6] with the specifications listed in Table S2. The octonary test set with only *O and *OH was calculated using the same specifications with GPAW version 23.9.1.

Table S1: The placed sites adsorbates' and the expression of their gas phase reference.

Adsorbate	Site	ΔE_{gas} formula
H	fcc	$\frac{1}{2}E_{H_2}$
O	fcc	$E_{H_2O} - E_{H_2}$
C	hcp	$E_{CH_4} - 2E_{H_2}$
OH	on-top	$E_{H_2O} - \frac{1}{2}E_{H_2}$
CH	hcp	$E_{CH_4} - \frac{3}{2}E_{H_2}$
NO	fcc	E_{NO}
CO	ont-top	E_{CO}
CH_2	hcp	$E_{CH_4} - E_{H_2}$
CH_3	on-top	$E_{CH_4} - \frac{1}{2}E_{H_2}$

Table S2: DFT specifications for adsorption energy calculations

Name	Specification
Calculator	GPAW version 22.1.0
Mode	Plane waves
Energy cutoff	400 eV
Brillouin zone sampling	Monkhorst-Pack grid
k-points	(4,4,1)
Functional	RPBE
Optimizer	LBFGS
Max force criteria	0.1 eV/Å

Structures with the adsorbate dissociated or desorbed as well as structures that were not converged by the criterium, were disregarded resulting in a total of 28113 adsorption energies on 1920 converged unique slabs in the main HEA dataset. The verified HEA dataset was split according to the slab numbers by an 80/10/10 training/validation/test split, assigning all calculations of the same slab, to the same set, to prevent data spillage. The number of slab- and adsorbate structures, including for each adsorbate, in each dataset are listed in Table S3 of the SI.

Table S3: Number of unique slabs and adsorption energies for each adsorbates in the datasets used for training, validating, and testing the finetuned model.

Dataset	slabs	H	O	C	OH	CH	NO	CO	CH2	CH3	total ads
train HEA	1536	2750	2465	2860	2260	2771	1594	2607	2294	2877	22478
val HEA	192	342	299	347	271	341	208	334	276	364	2782
test HEA	192	351	316	367	291	341	192	341	291	363	2853
test pure	12	12	12	12	12	12	12	12	12	12	108
test binary	63	124	122	124	116	122	97	118	107	124	1054
test ternary	211	411	378	403	354	407	261	398	340	412	3364
test octonary	100	N/A	187	N/A	182	N/A	N/A	N/A	N/A	N/A	369

S1.2 Fine-tuning of Open Catalyst Project Adsorption Model

The fine-tuning was run for 200 epochs saving the state at the epoch scoring the lowest MAE on the validation set. The fine-tuning process is described in further detail in Clausen et al.[3] and detailed configurations are listed in Table S4.

Table S4: Configuration for training, model architecture, and optimization settings.

Category	Value
**Training and Validation**	
Training Loss Function	MSE
Cross-Validation Metric	MAE of energy
**Model**	
name	equiformer_v2
use_pbc	True
regress_forces	True
otf_graph	True
max_neighbors	20
max_radius	12.0
max_num_elements	90
num_layers	8
sphere_channels	128
attn_hidden_channels	64
num_heads	8
attn_alpha_channels	64
attn_value_channels	16
ffn_hidden_channels	128
norm_type	layer_norm_sh
lmax_list	[4]
mmax_list	[2]
grid_resolution	18
num_sphere_samples	128
edge_channels	128
use_atom_edge_embedding	True
distance_function	gaussian
num_distance_basis	512
attn_activation	silu
use_s2_act_attn	False
ffn_activation	silu
use_gate_act	False
use_grid_mlp	True
alpha_drop	0.1
drop_path_rate	0.1
proj_drop	0.0
weight_init	uniform
**Optimization**	

train batch size	16
eval batch size	8
load balancing	atoms
num workers	8
lr initial	0.0012
optimizer	AdamW
weight decay	0.001
lr scheduler	ReduceLROnPlateau
lr scheduler mode	min
lr scheduler factor	0.8
lr scheduler patience	3
max epochs	200
clip_grad_norm	100
ema_decay	0.999

S1.3 Objectives

This demonstrative work utilizes two previously published simulation models. The catalytic ORR activity for a given composition is obtained from the model described in Clausen et al. [7], simulating a 96x96x5 fcc(111) surrogate surface. In this work, the *O and *OH adsorption energies of the surrogate surface are predicted using the fine-tuned OCP-model on 3x3x5 substructures of the surrogate surface for their assumed adsorption sites (fcc and on-top, respectively). The adsorption energies are adjusted to be in reference to the predicted adsorption energies on pristine Pt(111). The surface is covered by adsorbates from the lowest- to highest adsorption energy while adhering to a set of blocking rules to obtain a net adsorption energy distribution [8]. The activity is calculated as an average diffusion-limited current density of each site using the equations described in Clausen et al [7].

The operational stability of a composition is obtained through the dissolution simulation fully described in Plenge et al. [9] The simulation iteratively predicts individual dissolution potentials of surface atoms and subsequently removes atoms with dissolution potential below the applied potential. The fraction of remaining (111) surface atoms before ($N_{(111)}^{initial}$) versus after ($N_{(111)}^{final}$) dissolution is used as the stability parameter: $S_d = N_{(111)}^{final}/N_{(111)}^{initial}$. The parameter is calculated across 10 randomly initiated 4 nm truncated octahedron fcc particles (1925 atoms) with the given composition.

In both objectives an applied potential of 0.8 V vs. the reversible hydrogen electrode is used, providing great room for improvement in activity relative to Pt with an *OH energy 0.1 eV below the optimal *OH binding energy of 0.86 eV [7,10]. However, Pt will be deemed fully stable against dissolution by the applied model, for which unary compositions obtain a binary 0 or 1 in stability.

Finally, a third objective relating to the cost of the alloy composition is implemented. The price of each element is calculated and presented in $/mol in Table S5. For consistency with maximization, the objective is defined as a discount relative to the most expensive element in $/mol included in our analysis, namely Iridium: Discount $= p_{max} - p(x_{alloy})$.

Table S5: Prices of the individual metals. The prices per weight are obtained from `https://www.dailymetalprice.com/metalprices.php` on 16-06-2025. The molar wights are obtained from [11]. The molar price is given by price × molar weight.

Metal	Price [$/g]	Molar weight [g/mol]	Molar price [$/mol]
Ag	1.1684	107.87	126.035308
Au	109.09	196.97	21487.4573
Cu	0.01062	63.546	0.67485852
Ir	136.64	192.217	26264.53088
Pd	33.196	106.42	3532.71832
Pt	39.934	195.08	7790.32472
Rh	179.24	102.905	18444.6922
Ru	21.702	101.07	2193.42114

S1.4 Multi-Objective Bayesian Optimization

The standard performance metric in multi-objective optimization is the spanned hypervolume (HV) of the Pareto front relative to a reference point. An increase in HV thus indicates an expansion of the Pareto front through the discovery of a new Pareto optimal, or an improvement with respect to preliminary Pareto optima. Thus, maximizing the HV is a straightforward choice as an optimization objective in multi-objective Bayesian optimization (MOBO), which can be achieved through expected hypervolume improvement (EHVI) acquisition functions[12]. The Gaussian processes (GPs) and acquisition functions are implemented using the BoTorch module[13]. We employ a noisy expected hypervolume improvement acquisition function, *qLogNoisyExpectedHypervolumeImprovement*[14,15] (qlogNEHVI), which leverages Monte Carlo integration to account for noise in the observed samples when calculating the expected hypervolume improvement. In the case of single-objective optimization of the activity we utilize the single-objective version *qLogNoisyExpectedImprovement* (qLogNEI)[13,14]. Each non-deterministic objective (activity and stability) was modeled by independent GPs, both employing a scaled RBF kernel with a length scale for each dimension, referred to as ARD (automatic relevance determination) length scales, and homoscedastic inferred noise. The cost objective was calculated directly based on the composition.

In calculating the acquisition function the Monte Carlo integration is constrained to only consider feasible objective values for the activity and stability objectives, being between 0 and 0.608 for activity and between 0 and 1 for stability. The maximum activity is the activity obtained from all sites having the optimal adsorption energy at a coverage of 2/3 ML, which is the maximum assumed coverage[7].

The acquisition function is optimized through a simplex grid optimization scheme adopted from Pedersen et al.[16] and modified to include multiple restarts. The grid optimization implicitly ensures the composition constraints without using constrained optimization. The optimizer is described by the following scheme:

1. A compositional grid X_{grid} is created with step size d.
2. The acquisition function ($acqf$) is evaluated at the grid points: $y_{grid} = acqf(X_{grid})$
3. The $N_{restarts}$ best grid points is obtained: $X_{grid,best}$
4. Starting from each point x with corresponding y in $X_{grid,best}$, the acqf. is further optimized:
   (a) Step size is halved: $d = d/2$
   (b) Get surrounding compositions with grid step d: X_{around}
   (c) Get acqf. values: $y_{around} = acqf(X_{around})$.
   (d) Set x equal to best X_{around} if any $y_{around} > y$
   (e) If $d \leq$step threshold, save x and y in lists. Otherwise, repeat from (a).
5. Return the best candidate found from the $N_{restarts}$

In this work, we employed an initial grid size of 10 atomic percent (at. %) and continue until a step-size threshold of 0.5 at. % is reached.

The MOBO of the octonary space is initiated with eight pure metals and 20 random HEA compositions. The random samples are drawn from Dirichlet distributions: 10 from a uniform distribution ($\alpha = 1$) and 10 from an edge-favoring distribution ($\alpha = 0.2$). The optimization is run to provide 250 additional samples to construct an estimation of the Pareto front. In the subsystems, the samples from the higher dimensional spaces that fall within the subspace are used, along with a new set of 20 random samples, as the initial samples, from which MOBO is run for 100 iterations.

S2 Results

S2.1 Adsorption Energy Fine-tuning

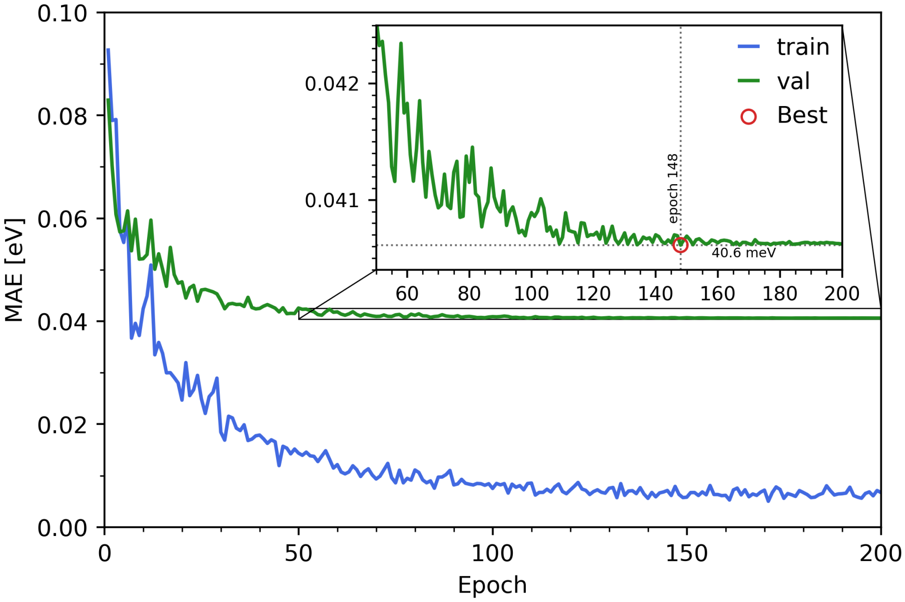

Figure S1: Learning curve for fine-tuning showing training and validation MAE loss per epoch.

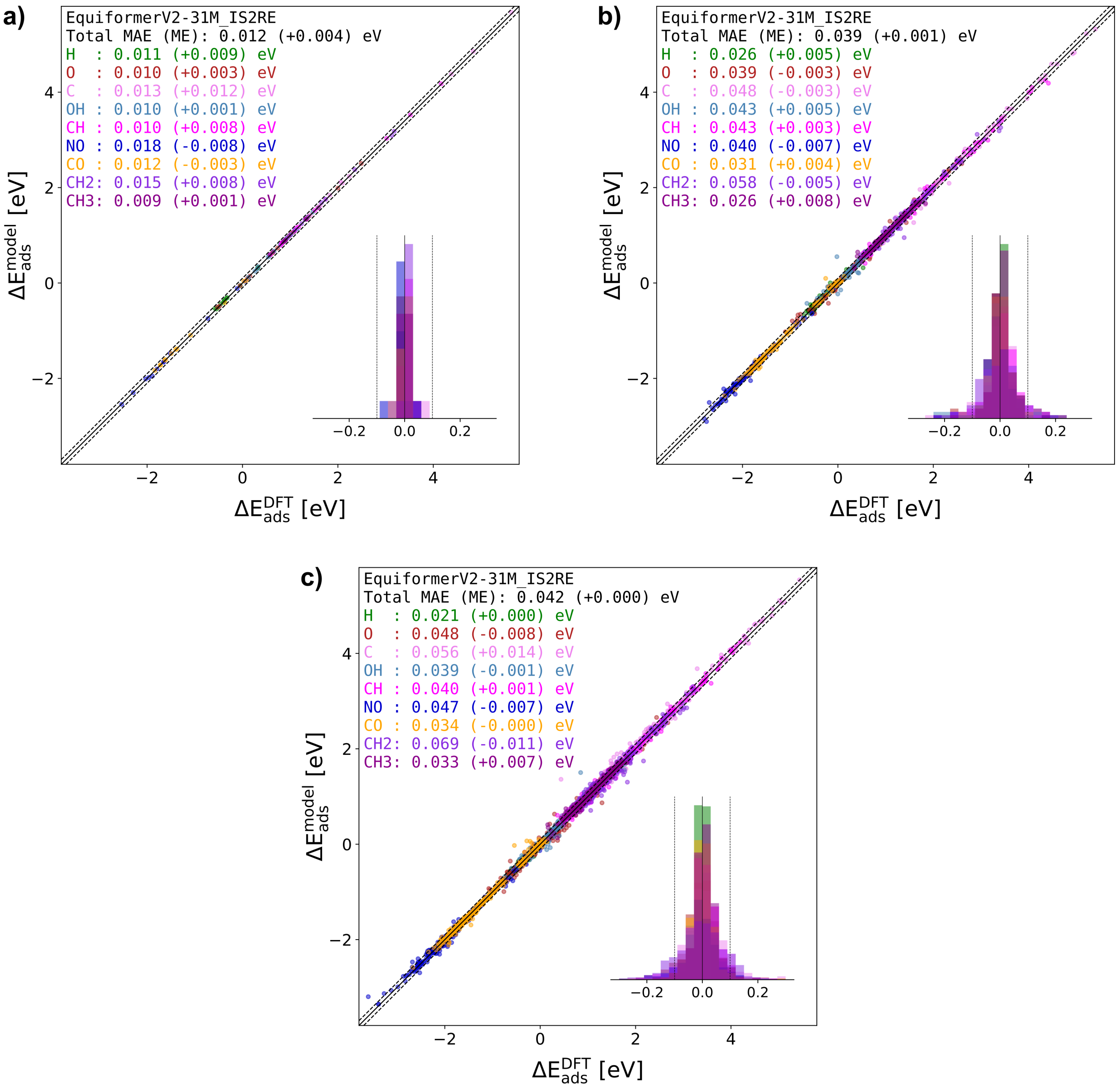

Figure S2: Adsorption energy regression parity plots for low-entropic test sets for a) single metals, b) binary alloys, and c) ternary alloys.

S2.2 Multi-Objective Bayesian Optimization

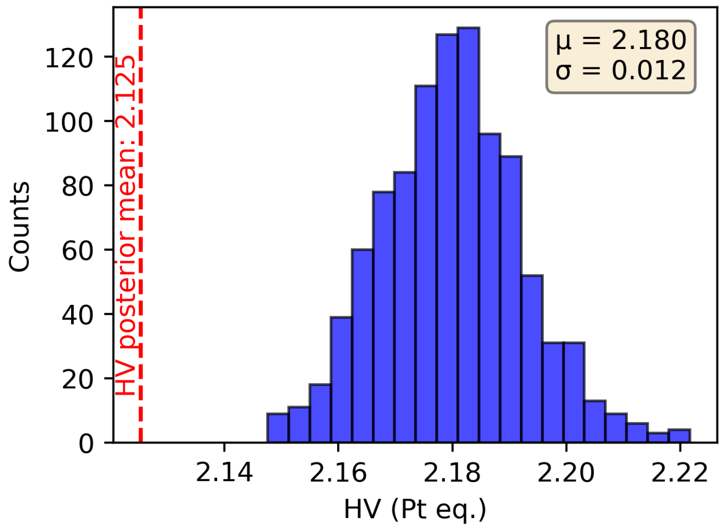

Figure S3: Distribution of computed HVs by adding the inferred noise levels to the posterior means on all sampled compositions. The applied noise levels of 0.023 and 0.033 (standard deviations in Pt. eq.) are obtained from the Gaussian likelihoods of the activity and stability GPs, respectively, fitted to all sampled compositions. The distribution contains 1000 HVs, where each is computed from randomly sampled noise from a normal distribution with the specified noise levels added to the mean of the posterior at each sampled composition. The stability objective is capped at 1. The cost objective is kept noiseless.

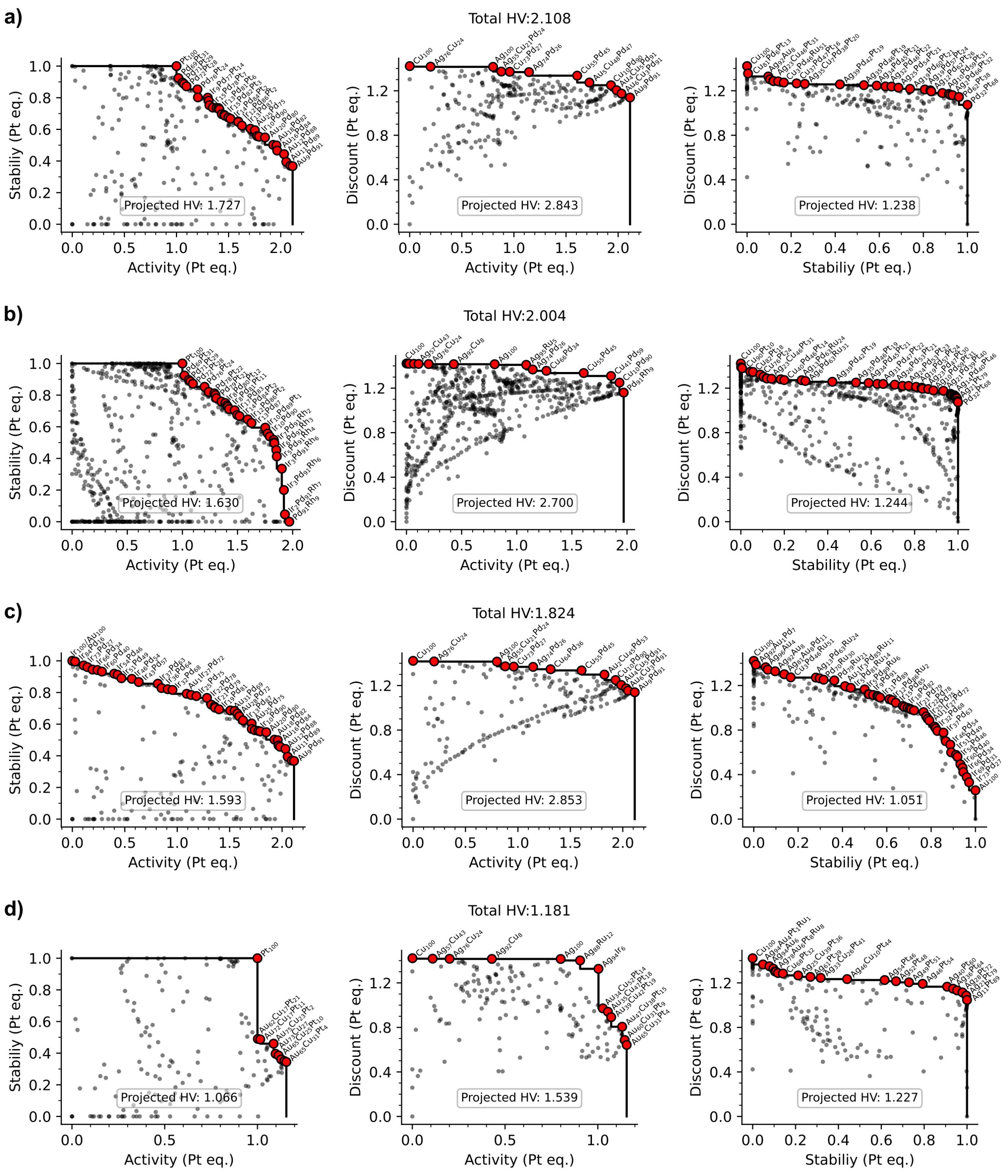

Figure S4: Projected Pareto fronts obtained from the individual MOBO runs: a) Ag-Au-Cu-Ir-Pd-Pt-Rh-Ru, b) Ag-Cu-Ir-Pd-Pt-Rh-Ru, c) Ag-Au-Cu-Ir-Pd-Rh-Ru, d) Ag-Au-Cu-Ir-Pt-Rh-Ru, e) Ag-Au-Cu-Ir-Rh-Ru, f) Ag-Cu-Ir-Rh-Ru, g) Ag-Au-Cu-Pd-Pt, and h) Ag-Au-Cu-Pd-Pt. The annotated Pareto optimal compositions is a representative selection. (Part 1)

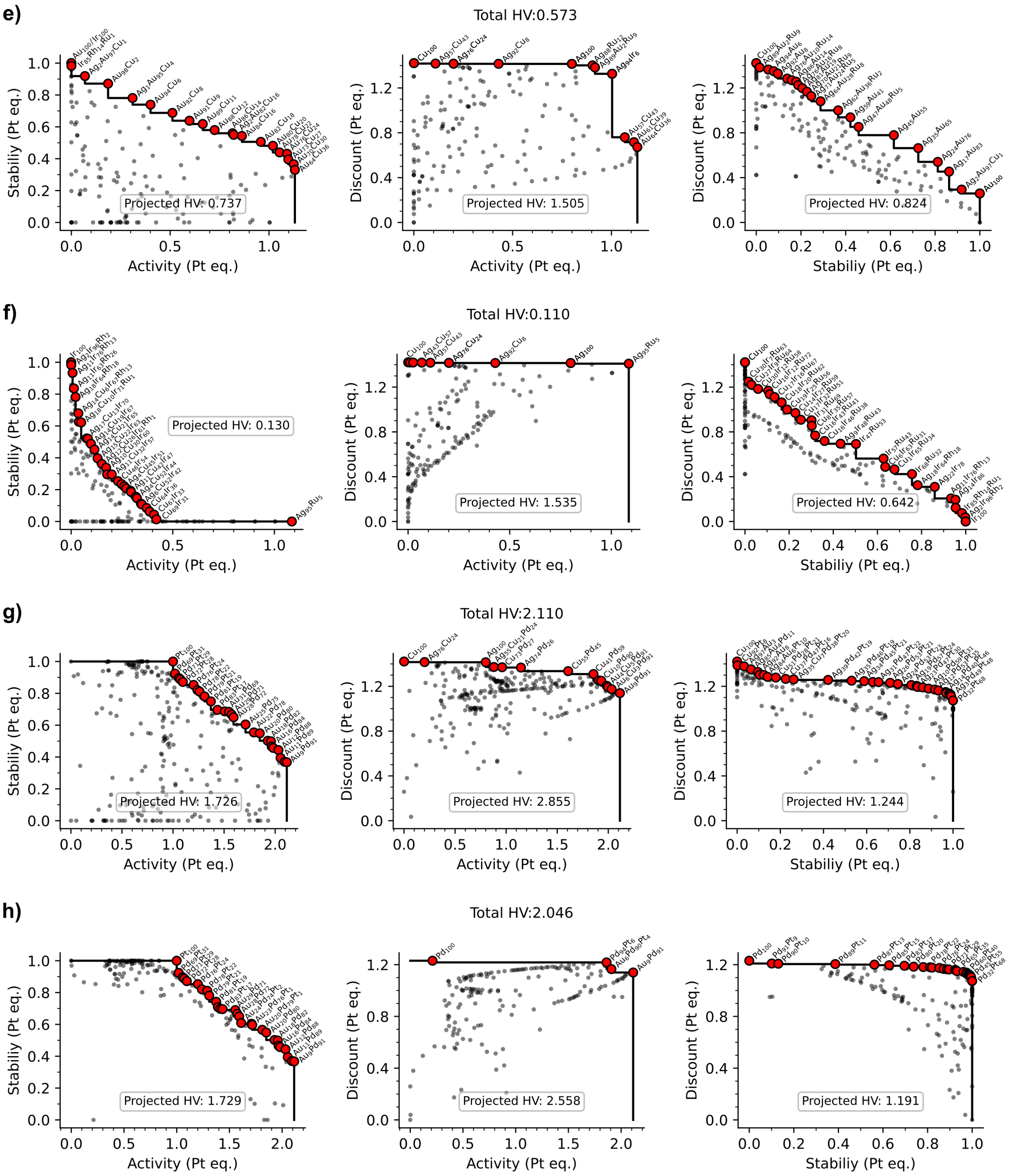

Figure S4: Projected Pareto fronts obtained from the individual MOBO runs: a) Ag-Au-Cu-Ir-Pd-Pt-Rh-Ru, b) Ag-Cu-Ir-Pd-Pt-Rh-Ru, c) Ag-Au-Cu-Ir-Pd-Rh-Ru, d) Ag-Au-Cu-Ir-Pt-Rh-Ru, e) Ag-Au-Cu-Ir-Rh-Ru, f) Ag-Cu-Ir-Rh-Ru, g) Ag-Au-Cu-Pd-Pt, and h) Ag-Au-Cu-Pd-Pt. The annotated Pareto optimal compositions is a representative selection. (Part 2)

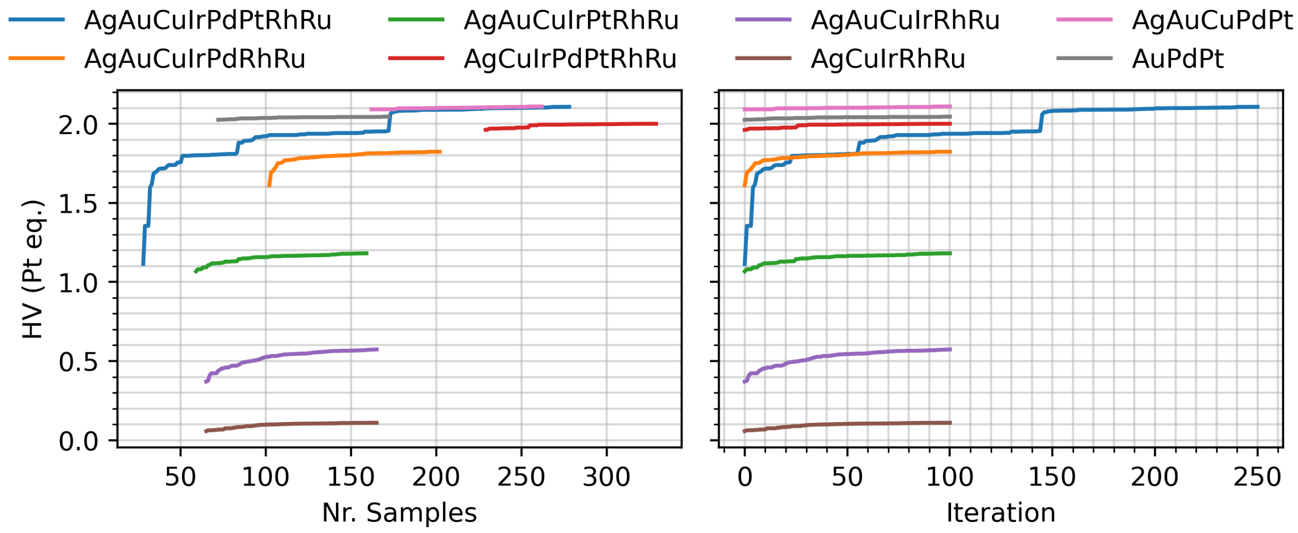

Figure S5: The HV for each run of MOBO as a function of number of samples (left) and iteration of MOBO (right). The subsystems are initiated from samples of higher systems that are within the respective subsystem with the addition of 20 random samples (10 uniform and 10 closer to the simplex edges). The three 7-element compositions and AgAuCuPdPt are initiated from the octonary space. AgAuCuIrRhRu are initiated from AgAuCuIrPdRhRu and AgAuCuIrPtRhRu, AgCuIrRhRu from AgAuCuIrRhRu, and AuPdPt from AgAuCuPdPt.

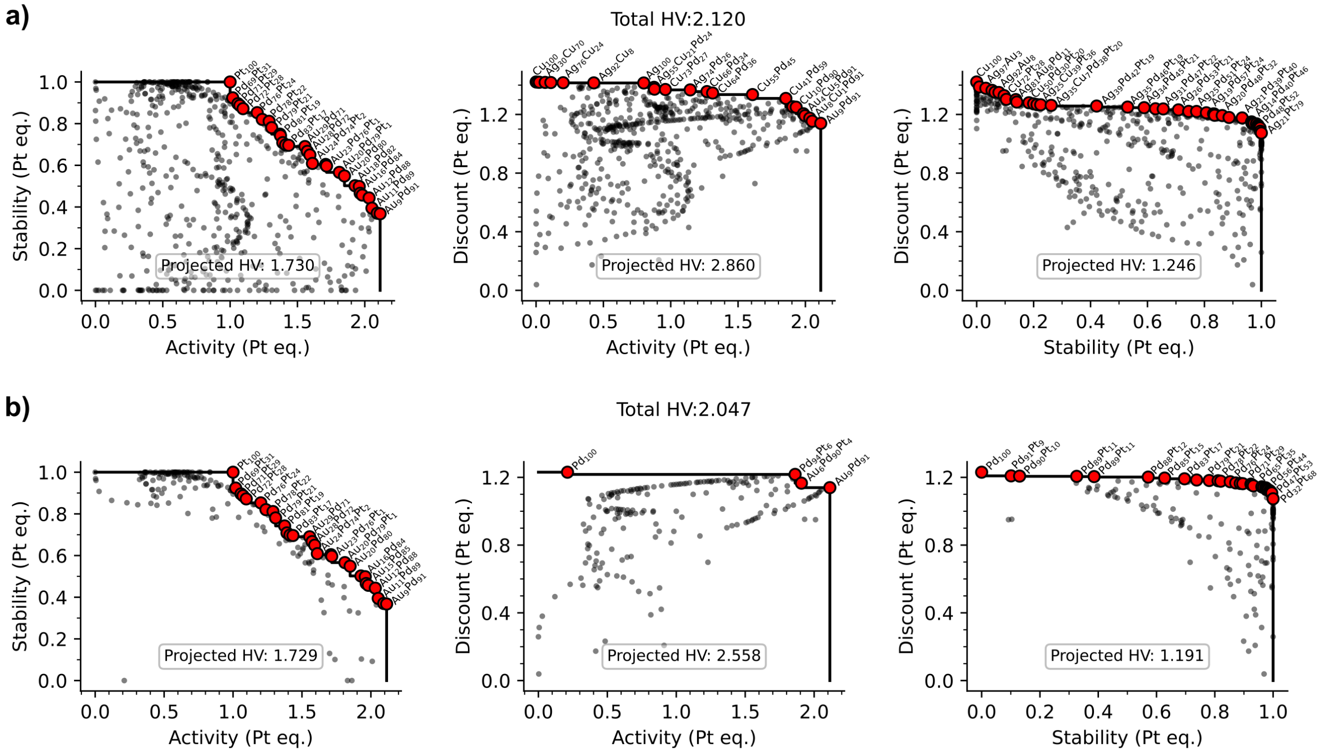

Figure S6: Projected Pareto fronts of critical subspaces obtained from all samples across all MOBO runs: a) Ag-Au-Cu-Pd-Pt and b) Au-Pd-Pt. The annotated Pareto optimal compositions is a representative selection.

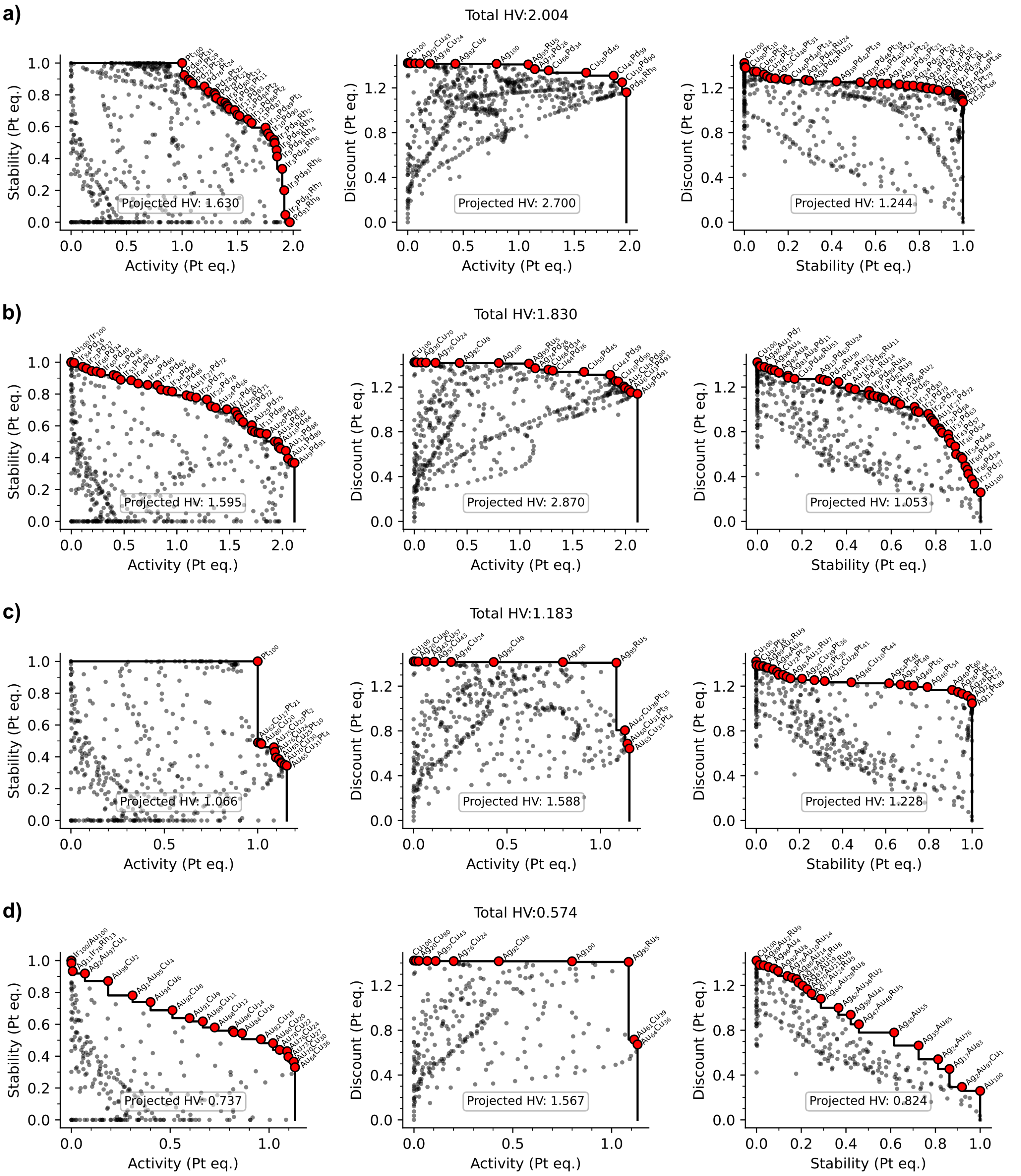

Figure S7: Projected Pareto fronts with removed critical elements obtained from the all samples across MOBO runs: a) Ag-Cu-Ir-Pd-Pt-Rh-Ru, b) Ag-Au-Cu-Ir-Pd-Rh-Ru, c) Ag-Au-Cu-Ir-Pt-Rh-Ru, d) Ag-Au-Cu-Ir-Rh-Ru, e) Ag-Cu-Ir-Rh-Ru. The annotated Pareto optimal compositions is a representative selection. (Part 1)

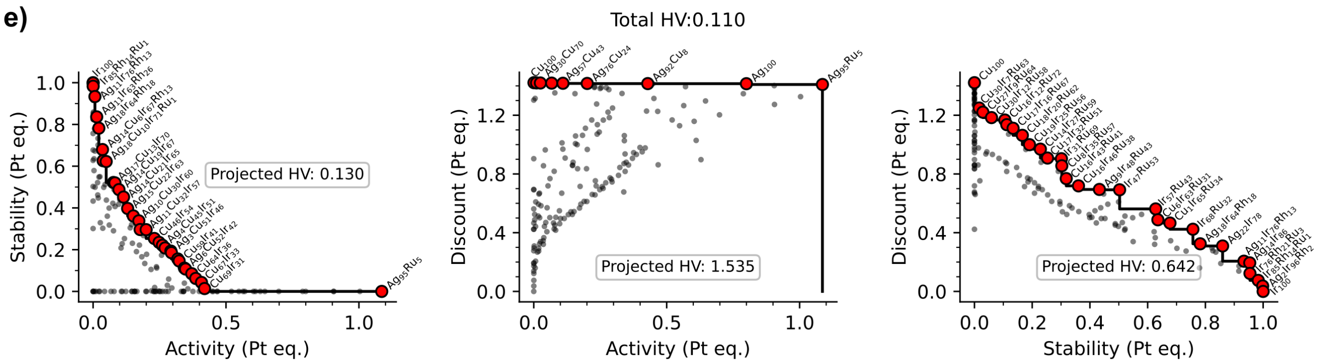

Figure S7: Projected Pareto fronts with removed critical elements obtained from the all samples across MOBO runs: a) Ag-Cu-Ir-Pd-Pt-Rh-Ru, b) Ag-Au-Cu-Ir-Pd-Rh-Ru, c) Ag-Au-Cu-Ir-Pt-Rh-Ru, d) Ag-Au-Cu-Ir-Rh-Ru, e) Ag-Cu-Ir-Rh-Ru. The annotated Pareto optimal compositions is a representative selection. (Part 2)

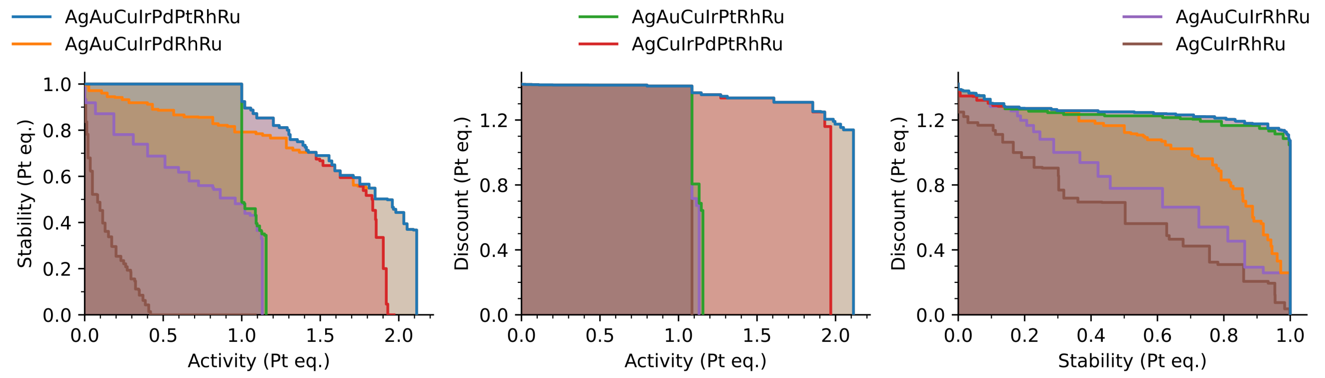

Figure S8: The projected Pareto fronts containing all data within respective system overlayed for comparison.

Table S6: All the found Pareto optimal compositions clustered using a Gaussian mixture model using the number of clusters yielding the lowest Bayesian information criterion. The clustering is performed only on the compositions, which were transformed with an isometric log-ratio where 0.0 were set to 0.1 times the smallest non-zero value across all compositions, and subsequently standardized. The clusters are named by their main constituting elements. The objectives are normalized by Pt.

Cluster	Ag	Au	Cu	Ir	Pd	Pt	Rh	Ru	Act.	Stab.	Disc.
	0.0	0.094	0.0	0.0	0.906	0.0	0.0	0.0	2.114	0.367	1.139
	0.0	0.1	0.0	0.0	0.9	0.0	0.0	0.0	2.093	0.37	1.133
	0.0	0.106	0.0	0.0	0.894	0.0	0.0	0.0	2.053	0.395	1.127
	0.0	0.125	0.0	0.0	0.875	0.0	0.0	0.0	2.032	0.443	1.109
	0.0	0.15	0.0	0.0	0.85	0.0	0.0	0.0	1.981	0.457	1.085
	0.0	0.156	0.0	0.0	0.844	0.0	0.0	0.0	1.966	0.466	1.079
	0.0	0.162	0.0	0.0	0.838	0.0	0.0	0.0	1.959	0.498	1.073
Au-Pd	0.0	0.175	0.0	0.0	0.825	0.0	0.0	0.0	1.928	0.502	1.06
	0.0	0.2	0.0	0.0	0.8	0.0	0.0	0.0	1.849	0.549	1.036
	0.0	0.206	0.0	0.0	0.788	0.006	0.0	0.0	1.811	0.566	1.029
	0.0	0.231	0.0	0.0	0.763	0.006	0.0	0.0	1.716	0.597	1.004
	0.0	0.25	0.0	0.0	0.75	0.0	0.0	0.0	1.71	0.604	0.987
	0.0	0.275	0.0	0.0	0.725	0.0	0.0	0.0	1.593	0.65	0.963
	0.0	0.281	0.0	0.0	0.719	0.0	0.0	0.0	1.574	0.667	0.957
	0.0	0.287	0.0	0.0	0.713	0.0	0.0	0.0	1.556	0.69	0.951
	0.013	0.0	0.05	0.0	0.781	0.156	0.0	0.0	1.41	0.545	1.206
	0.05	0.0	0.131	0.0	0.644	0.175	0.0	0.0	1.264	0.461	1.224
	0.063	0.0	0.3	0.0	0.456	0.181	0.0	0.0	1.18	0.259	1.258
	0.15	0.0	0.031	0.0	0.644	0.175	0.0	0.0	1.144	0.626	1.224
Ag-Cu-Pd(-Pt)	0.15	0.0	0.1	0.0	0.594	0.156	0.0	0.0	1.162	0.412	1.241
	0.263	0.0	0.062	0.0	0.481	0.194	0.0	0.0	0.964	0.462	1.246
	0.275	0.0	0.056	0.0	0.475	0.194	0.0	0.0	0.963	0.486	1.247
	0.281	0.0	0.038	0.0	0.481	0.2	0.0	0.0	0.942	0.517	1.243
	0.3	0.019	0.244	0.0	0.437	0.0	0.0	0.0	1.267	0.024	1.314
	0.0	0.0	0.0	0.0	0.7	0.0	0.0	0.3	1.154	0.315	1.252
	0.0	0.0	0.0	0.0	0.787	0.0	0.0	0.213	1.398	0.363	1.246
	0.0	0.0	0.0	0.0	0.831	0.0	0.0	0.169	1.544	0.322	1.243
	0.0	0.019	0.0	0.0	0.9	0.0	0.0	0.081	1.809	0.249	1.218
Pd-Ru	0.0	0.038	0.0	0.0	0.875	0.0	0.0	0.087	1.766	0.341	1.2
	0.0	0.056	0.0	0.0	0.9	0.0	0.0	0.044	1.926	0.336	1.179
	0.063	0.0	0.0	0.0	0.625	0.0	0.0	0.312	1.022	0.297	1.265
	0.113	0.0	0.0	0.0	0.731	0.0	0.0	0.156	1.253	0.227	1.263
	0.125	0.0	0.0	0.0	0.631	0.0	0.0	0.244	1.033	0.28	1.271
	0.0	0.0	0.0	0.056	0.888	0.0	0.0	0.056	1.742	0.5	1.165
Ir-Pd-Ru	0.0	0.0	0.0	0.125	0.856	0.0	0.0	0.019	1.595	0.612	1.078
	0.0	0.006	0.0	0.031	0.85	0.0	0.0	0.113	1.6	0.412	1.194
	0.0	0.0	0.0	0.0	0.306	0.694	0.0	0.0	0.364	1.0	1.071
	0.0	0.0	0.0	0.0	0.319	0.681	0.0	0.0	0.361	1.0	1.073
	0.0	0.0	0.0	0.0	0.469	0.531	0.0	0.0	0.531	0.995	1.108
	0.0	0.0	0.0	0.0	0.475	0.525	0.0	0.0	0.548	0.993	1.109
Pd-Pt	0.0	0.0	0.0	0.0	0.5	0.5	0.0	0.0	0.572	0.991	1.115

	0.0	0.0	0.0	0.0	0.525	0.475	0.0	0.0	0.617	0.989	1.121
	0.0	0.0	0.0	0.0	0.55	0.45	0.0	0.0	0.67	0.983	1.127
	0.0	0.0	0.0	0.0	0.556	0.444	0.0	0.0	0.69	0.978	1.128
	0.0	0.0	0.0	0.0	0.569	0.431	0.0	0.0	0.712	0.977	1.131
	0.0	0.0	0.0	0.0	0.588	0.412	0.0	0.0	0.738	0.977	1.135
	0.0	0.0	0.0	0.0	0.6	0.4	0.0	0.0	0.771	0.974	1.138
	0.0	0.0	0.0	0.0	0.606	0.394	0.0	0.0	0.789	0.967	1.14
	0.0	0.0	0.0	0.0	0.625	0.375	0.0	0.0	0.838	0.961	1.144
	0.0	0.0	0.0	0.0	0.65	0.35	0.0	0.0	0.894	0.932	1.15
	0.0	0.0	0.0	0.0	0.675	0.325	0.0	0.0	0.962	0.927	1.156
	0.0	0.0	0.0	0.0	0.694	0.306	0.0	0.0	1.019	0.924	1.16
	0.0	0.0	0.0	0.0	0.706	0.294	0.0	0.0	1.058	0.896	1.163
	0.0	0.0	0.0	0.0	0.713	0.287	0.0	0.0	1.072	0.887	1.164
	0.0	0.0	0.0	0.0	0.725	0.275	0.0	0.0	1.096	0.872	1.167
	0.0	0.0	0.0	0.0	0.756	0.244	0.0	0.0	1.201	0.853	1.174
	0.0	0.0	0.0	0.0	0.769	0.231	0.0	0.0	1.239	0.82	1.177
	0.0	0.0	0.0	0.0	0.781	0.219	0.0	0.0	1.29	0.811	1.18
	0.0	0.0	0.0	0.0	0.787	0.213	0.0	0.0	1.309	0.781	1.181
	0.0	0.0	0.0	0.0	0.831	0.169	0.0	0.0	1.435	0.696	1.192
	0.0	0.0	0.0	0.0	0.85	0.15	0.0	0.0	1.505	0.628	1.196
	0.0	0.0	0.0	0.0	0.875	0.125	0.0	0.0	1.571	0.572	1.202
	0.0	0.0	0.0	0.0	0.887	0.113	0.0	0.0	1.64	0.386	1.205
	0.0	0.0	0.0	0.0	0.894	0.106	0.0	0.0	1.66	0.326	1.206
	0.925	0.075	0.0	0.0	0.0	0.0	0.0	0.0	0.482	0.096	1.328
	0.944	0.037	0.0	0.0	0.0	0.013	0.0	0.006	0.677	0.048	1.366
**Ag**(-Au)	0.944	0.056	0.0	0.0	0.0	0.0	0.0	0.0	0.53	0.078	1.35
	0.956	0.044	0.0	0.0	0.0	0.0	0.0	0.0	0.565	0.054	1.364
	0.969	0.031	0.0	0.0	0.0	0.0	0.0	0.0	0.602	0.032	1.379
	1.0	0.0	0.0	0.0	0.0	0.0	0.0	0.0	0.8	0.0	1.415
	0.006	0.0	0.0	0.0	0.806	0.188	0.0	0.0	1.371	0.749	1.188
	0.013	0.0	0.0	0.0	0.581	0.406	0.0	0.0	0.748	0.968	1.139
	0.019	0.0	0.0	0.0	0.769	0.212	0.0	0.0	1.276	0.773	1.185
	0.025	0.0	0.0	0.0	0.656	0.319	0.0	0.0	0.941	0.923	1.162
	0.031	0.0	0.0	0.0	0.494	0.475	0.0	0.0	0.621	0.988	1.127
	0.031	0.0	0.0	0.0	0.519	0.45	0.0	0.0	0.665	0.983	1.133
	0.05	0.0	0.0	0.0	0.769	0.181	0.0	0.0	1.272	0.693	1.198
	0.069	0.0	0.0	0.0	0.6	0.331	0.0	0.0	0.899	0.916	1.167
	0.081	0.0	0.0	0.0	0.613	0.306	0.0	0.0	0.963	0.879	1.175
	0.094	0.0	0.0	0.0	0.712	0.194	0.0	0.0	1.202	0.729	1.203
	0.094	0.0	0.0	0.0	0.744	0.162	0.0	0.0	1.283	0.682	1.21
	0.106	0.0	0.0	0.0	0.588	0.306	0.0	0.0	0.949	0.886	1.179
	0.112	0.0	0.0	0.0	0.388	0.5	0.0	0.0	0.6	0.983	1.136
	0.112	0.0	0.0	0.0	0.494	0.394	0.0	0.0	0.79	0.932	1.16
	0.119	0.0	0.0	0.0	0.531	0.35	0.0	0.0	0.856	0.916	1.172
	0.125	0.0	0.0	0.0	0.594	0.281	0.0	0.0	0.958	0.855	1.189
	0.125	0.0	0.0	0.0	0.619	0.256	0.0	0.0	1.014	0.836	1.194
Ag-Pd-Pt	0.137	0.0	0.0	0.0	0.4	0.463	0.0	0.0	0.661	0.966	1.149

	0.15	0.0	0.0	0.0	0.556	0.294	0.0	0.0	0.936	0.86	1.19
	0.163	0.0	0.0	0.0	0.662	0.175	0.0	0.0	1.124	0.702	1.22
	0.169	0.0	0.0	0.0	0.581	0.25	0.0	0.0	1.015	0.806	1.204
	0.181	0.0	0.0	0.0	0.556	0.263	0.0	0.0	0.963	0.834	1.203
	0.188	0.0	0.0	0.0	0.606	0.206	0.0	0.0	1.067	0.74	1.218
	0.194	0.0	0.0	0.0	0.569	0.237	0.0	0.0	1.0	0.807	1.211
	0.2	0.0	0.0	0.0	0.475	0.325	0.0	0.0	0.848	0.862	1.192
	0.206	0.0	0.0	0.0	0.394	0.4	0.0	0.0	0.697	0.933	1.176
	0.231	0.0	0.0	0.0	0.469	0.3	0.0	0.0	0.844	0.832	1.204
	0.231	0.0	0.0	0.0	0.519	0.25	0.0	0.0	0.912	0.756	1.215
	0.231	0.0	0.0	0.0	0.6	0.169	0.0	0.0	1.046	0.652	1.234
	0.238	0.0	0.0	0.0	0.531	0.231	0.0	0.0	0.944	0.745	1.221
	0.25	0.0	0.0	0.0	0.506	0.244	0.0	0.0	0.895	0.772	1.22
	0.25	0.0	0.0	0.0	0.569	0.181	0.0	0.0	0.994	0.617	1.235
	0.263	0.0	0.0	0.0	0.531	0.206	0.0	0.0	0.962	0.709	1.231
	0.312	0.0	0.0	0.0	0.469	0.219	0.0	0.0	0.906	0.655	1.238
	0.319	0.0	0.0	0.0	0.462	0.219	0.0	0.0	0.899	0.628	1.239
	0.344	0.0	0.0	0.0	0.45	0.206	0.0	0.0	0.9	0.588	1.246
	0.344	0.0	0.0	0.0	0.462	0.194	0.0	0.0	0.922	0.508	1.249
	0.344	0.0	0.0	0.0	0.481	0.175	0.0	0.0	0.965	0.374	1.254
	0.35	0.0	0.0	0.0	0.456	0.194	0.0	0.0	0.921	0.53	1.25
	0.369	0.0	0.006	0.0	0.425	0.2	0.0	0.0	0.89	0.416	1.254
	0.387	0.0	0.0	0.0	0.419	0.194	0.0	0.0	0.883	0.421	1.257
Ir-Pd-Rh	0.0	0.0	0.0	0.05	0.912	0.0	0.038	0.0	1.856	0.413	1.139
	0.0	0.0	0.0	0.056	0.913	0.0	0.031	0.0	1.852	0.453	1.136
	0.0	0.0	0.0	0.069	0.912	0.0	0.019	0.0	1.834	0.496	1.131
	0.0	0.0	0.0	0.075	0.912	0.0	0.013	0.0	1.833	0.501	1.128
Cu-Pd-Pt	0.0	0.0	0.081	0.0	0.781	0.138	0.0	0.0	1.491	0.457	1.214
	0.0	0.0	0.087	0.0	0.8	0.113	0.0	0.0	1.579	0.362	1.221
	0.0	0.0	0.131	0.0	0.744	0.125	0.0	0.0	1.489	0.402	1.227
	0.0	0.0	0.225	0.0	0.631	0.144	0.0	0.0	1.413	0.333	1.24
	0.0	0.0	0.25	0.0	0.644	0.106	0.0	0.0	1.517	0.153	1.254
	0.0	0.0	0.263	0.0	0.606	0.131	0.0	0.0	1.439	0.302	1.25
	0.0	0.0	0.3	0.0	0.525	0.175	0.0	0.0	1.294	0.314	1.247
	0.0	0.0	0.375	0.0	0.469	0.156	0.0	0.0	1.333	0.225	1.266
	0.0	0.0	0.394	0.0	0.462	0.144	0.0	0.0	1.355	0.2	1.273
	0.0	0.0	0.394	0.0	0.469	0.137	0.0	0.0	1.37	0.176	1.274
	0.0	0.0	0.406	0.0	0.475	0.119	0.0	0.0	1.42	0.083	1.281
	0.0	0.0	0.481	0.0	0.363	0.156	0.0	0.0	1.23	0.12	1.286
	0.0	0.0	0.5	0.0	0.3	0.2	0.0	0.0	1.108	0.184	1.28
	0.0	0.0	0.0	0.0	0.0	0.994	0.0	0.006	0.873	0.965	1.002
	0.0	0.0	0.0	0.0	0.0	1.0	0.0	0.0	1.0	1.0	1.0
	0.0	0.0	0.0	0.0	0.006	0.994	0.0	0.0	0.833	1.0	1.001
	0.0	0.0	0.0	0.0	0.012	0.988	0.0	0.0	0.765	1.0	1.003
	0.0	0.0	0.0	0.0	0.037	0.963	0.0	0.0	0.615	1.0	1.009
	0.0	0.0	0.0	0.0	0.069	0.931	0.0	0.0	0.531	1.0	1.016
**Pt**(-Ag)	0.012	0.0	0.0	0.0	0.0	0.988	0.0	0.0	0.742	1.0	1.005

	0.013	0.0	0.0	0.0	0.006	0.981	0.0	0.0	0.698	1.0	1.007
	0.019	0.0	0.0	0.0	0.0	0.981	0.0	0.0	0.691	1.0	1.008
	0.031	0.0	0.0	0.0	0.0	0.969	0.0	0.0	0.61	1.0	1.013
	0.062	0.0	0.0	0.0	0.0	0.938	0.0	0.0	0.497	1.0	1.026
	0.113	0.0	0.0	0.0	0.0	0.887	0.0	0.0	0.404	1.0	1.047
	0.206	0.0	0.0	0.0	0.0	0.794	0.0	0.0	0.306	0.997	1.086
Au-Cu-Pd	0.0	0.038	0.056	0.0	0.906	0.0	0.0	0.0	1.986	0.161	1.205
	0.0	0.05	0.05	0.0	0.9	0.0	0.0	0.0	1.997	0.21	1.191
	0.0	0.063	0.025	0.0	0.912	0.0	0.0	0.0	2.032	0.281	1.174
	0.0	0.075	0.019	0.0	0.906	0.0	0.0	0.0	2.028	0.299	1.161
	0.0	0.081	0.013	0.0	0.906	0.0	0.0	0.0	2.051	0.341	1.154
	0.0	0.087	0.05	0.0	0.863	0.0	0.0	0.0	1.974	0.299	1.155
	0.1	0.037	0.144	0.0	0.719	0.0	0.0	0.0	1.636	0.103	1.24
Ir-Pd-Pt	0.0	0.0	0.0	0.006	0.813	0.181	0.0	0.0	1.383	0.735	1.181
	0.0	0.0	0.0	0.019	0.9	0.081	0.0	0.0	1.729	0.344	1.189
	0.0	0.0	0.0	0.031	0.625	0.344	0.0	0.0	0.855	0.94	1.113
	0.0	0.0	0.0	0.044	0.862	0.094	0.0	0.0	1.564	0.594	1.155
	0.0	0.0	0.0	0.044	0.887	0.069	0.0	0.0	1.695	0.519	1.161
	0.0	0.0	0.0	0.069	0.794	0.137	0.0	0.0	1.354	0.759	1.114
	0.0	0.0	0.0	0.075	0.8	0.125	0.0	0.0	1.39	0.75	1.109
	0.0	0.0	0.0	0.075	0.806	0.119	0.0	0.0	1.412	0.726	1.111
	0.0	0.0	0.0	0.081	0.813	0.106	0.0	0.0	1.424	0.712	1.106
	0.0	0.0	0.0	0.081	0.856	0.063	0.0	0.0	1.577	0.64	1.116
	0.0	0.0	0.0	0.087	0.775	0.138	0.0	0.0	1.3	0.8	1.091
	0.0	0.0	0.0	0.088	0.906	0.006	0.0	0.0	1.79	0.538	1.121
	0.0	0.0	0.0	0.094	0.894	0.012	0.0	0.0	1.735	0.565	1.112
	0.0	0.0	0.0	0.094	0.9	0.006	0.0	0.0	1.769	0.548	1.114
	0.0	0.0	0.0	0.1	0.894	0.006	0.0	0.0	1.752	0.595	1.106
	0.0	0.0	0.0	0.113	0.825	0.062	0.0	0.0	1.465	0.686	1.078
	0.0	0.0	0.0	0.119	0.856	0.025	0.0	0.0	1.593	0.647	1.079
	0.0	0.0	0.0	0.131	0.806	0.063	0.0	0.0	1.404	0.735	1.055
	0.0	0.0	0.0	0.144	0.837	0.019	0.0	0.0	1.517	0.667	1.049
	0.0	0.0	0.0	0.15	0.819	0.031	0.0	0.0	1.458	0.691	1.039
	0.0	0.0	0.0	0.15	0.831	0.019	0.0	0.0	1.497	0.675	1.042
	0.0	0.0	0.006	0.056	0.913	0.025	0.0	0.0	1.823	0.46	1.157
Ag-Cu	0.2	0.0	0.8	0.0	0.0	0.0	0.0	0.0	0.01	0.0	1.42
	0.3	0.0	0.7	0.0	0.0	0.0	0.0	0.0	0.025	0.0	1.42
	0.431	0.0	0.569	0.0	0.0	0.0	0.0	0.0	0.067	0.0	1.419
	0.575	0.0	0.425	0.0	0.0	0.0	0.0	0.0	0.11	0.0	1.418
	0.756	0.0	0.244	0.0	0.0	0.0	0.0	0.0	0.201	0.0	1.417
	0.919	0.0	0.081	0.0	0.0	0.0	0.0	0.0	0.429	0.0	1.415
Ag-Ru	0.831	0.069	0.0	0.0	0.0	0.0	0.0	0.1	0.717	0.094	1.324
	0.869	0.05	0.0	0.0	0.0	0.0	0.0	0.081	0.819	0.062	1.348
	0.887	0.019	0.0	0.0	0.0	0.0	0.0	0.094	0.917	0.015	1.383
	0.95	0.0	0.0	0.0	0.0	0.0	0.0	0.05	1.086	0.0	1.409
Ir-Pd	0.0	0.0	0.0	0.088	0.912	0.0	0.0	0.0	1.829	0.517	1.123
	0.0	0.0	0.0	0.094	0.906	0.0	0.0	0.0	1.792	0.539	1.115

	0.0	0.0	0.0	0.1	0.9	0.0	0.0	0.0	1.774	0.557	1.107
	0.0	0.0	0.0	0.131	0.869	0.0	0.0	0.0	1.627	0.624	1.069
	0.0	0.0	0.0	0.169	0.831	0.0	0.0	0.0	1.474	0.704	1.023
	0.0	0.0	0.019	0.062	0.919	0.0	0.0	0.0	1.848	0.419	1.157
	0.0	0.056	0.0	0.1	0.844	0.0	0.0	0.0	1.641	0.603	1.053
**Cu**(-Pt)	0.0	0.0	0.675	0.0	0.0	0.325	0.0	0.0	0.845	0.141	1.285
	0.0	0.0	0.719	0.0	0.0	0.281	0.0	0.0	0.814	0.104	1.303
	0.0	0.0	0.762	0.0	0.0	0.238	0.0	0.0	0.799	0.09	1.322
	0.0	0.0	1.0	0.0	0.0	0.0	0.0	0.0	0.001	0.0	1.422
Cu-Pd	0.0	0.0	0.1	0.0	0.9	0.0	0.0	0.0	1.931	0.0	1.25
	0.0	0.0	0.412	0.0	0.588	0.0	0.0	0.0	1.855	0.0	1.309
	0.0	0.0	0.55	0.0	0.45	0.0	0.0	0.0	1.607	0.0	1.336
	0.0	0.0	0.656	0.0	0.344	0.0	0.0	0.0	1.269	0.0	1.356
	0.0	0.006	0.638	0.0	0.356	0.0	0.0	0.0	1.311	0.001	1.346
	0.0	0.019	0.45	0.0	0.531	0.0	0.0	0.0	1.825	0.016	1.298
	0.0	0.019	0.556	0.0	0.425	0.0	0.0	0.0	1.566	0.013	1.319
	0.0	0.031	0.444	0.0	0.525	0.0	0.0	0.0	1.808	0.044	1.285
	0.0	0.037	0.119	0.0	0.844	0.0	0.0	0.0	1.908	0.114	1.217
	0.0	0.038	0.581	0.0	0.381	0.0	0.0	0.0	1.467	0.032	1.305
	0.0	0.044	0.337	0.0	0.619	0.0	0.0	0.0	1.907	0.078	1.252
	0.0	0.05	0.481	0.0	0.469	0.0	0.0	0.0	1.724	0.064	1.274
	0.0	0.063	0.356	0.0	0.581	0.0	0.0	0.0	1.874	0.118	1.238
	0.0	0.081	0.219	0.0	0.7	0.0	0.0	0.0	1.888	0.182	1.193
Ag-Au-Pd	0.619	0.037	0.0	0.0	0.344	0.0	0.0	0.0	1.352	0.084	1.308
	0.675	0.062	0.0	0.0	0.263	0.0	0.0	0.0	1.169	0.117	1.294
	0.694	0.025	0.0	0.0	0.281	0.0	0.0	0.0	1.214	0.039	1.334
	0.738	0.031	0.0	0.0	0.231	0.0	0.0	0.0	1.086	0.051	1.336
	0.744	0.0	0.0	0.0	0.256	0.0	0.0	0.0	1.145	0.0	1.368
	0.744	0.056	0.0	0.0	0.2	0.0	0.0	0.0	0.972	0.093	1.313
	0.813	0.081	0.0	0.0	0.106	0.0	0.0	0.0	0.675	0.138	1.301
	0.819	0.025	0.0	0.0	0.156	0.0	0.0	0.0	0.846	0.028	1.357
	0.837	0.044	0.0	0.0	0.119	0.0	0.0	0.0	0.739	0.063	1.342
	0.856	0.038	0.0	0.0	0.106	0.0	0.0	0.0	0.713	0.064	1.352
	0.919	0.012	0.0	0.0	0.069	0.0	0.0	0.0	0.68	0.01	1.388
Au-Pd-Pt	0.0	0.006	0.0	0.0	0.538	0.456	0.0	0.0	0.649	0.987	1.119
	0.0	0.006	0.0	0.0	0.806	0.188	0.0	0.0	1.374	0.742	1.181
	0.0	0.019	0.0	0.0	0.806	0.175	0.0	0.0	1.391	0.709	1.172
	0.0	0.019	0.0	0.0	0.812	0.169	0.0	0.0	1.413	0.698	1.173
	0.0	0.019	0.0	0.0	0.825	0.156	0.0	0.0	1.47	0.639	1.176
	0.0	0.025	0.0	0.0	0.906	0.069	0.0	0.0	1.845	0.334	1.19
	0.0	0.031	0.0	0.0	0.831	0.138	0.0	0.0	1.509	0.635	1.168
	0.0	0.031	0.0	0.0	0.85	0.119	0.0	0.0	1.583	0.516	1.173
	0.0	0.031	0.0	0.0	0.875	0.094	0.0	0.0	1.689	0.468	1.178
	0.0	0.088	0.0	0.0	0.812	0.1	0.0	0.0	1.585	0.53	1.122

References

[1] Y.-L. Liao, B. Wood, A. Das, T. Smidt, EquiformerV2: Improved Equivariant Transformer for Scaling to Higher-Degree Representations **2024**.

[2] L. Chanussot, A. Das, S. Goyal, T. Lavril, M. Shuaibi, M. Riviere, K. Tran, J. Heras-Domingo, C. Ho, W. Hu, A. Palizhati, A. Sriram, B. Wood, J. Yoon, D. Parikh, C. L. Zitnick, Z. Ulissi, *ACS Catalysis* **2021**, *11*, 6059.

[3] C. M. Clausen, J. Rossmeisl, Z. W. Ulissi, *The Journal of Physical Chemistry C* **2024**, *128*, 11190.

[4] A. Hjorth Larsen, J. Jørgen Mortensen, J. Blomqvist, I. E. Castelli, R. Christensen, M. Dułak, J. Friis, M. N. Groves, B. Hammer, C. Hargus, et al., *Journal of Physics: Condensed Matter* **2017**, *29*, 273002.

[5] C. M. Clausen, J. K. Pedersen, T. A. A. Batchelor, J. Rossmeisl, *Nano Research* **2022**, *15*, 4775–4779.

[6] J. Enkovaara, C. Rostgaard, J. J. Mortensen, J. Chen, M. Dułak, L. Ferrighi, J. Gavnholt, C. Glinsvad, V. Haikola, H. A. Hansen, et al., *Journal of Physics: Condensed Matter* **2010**, *22*, 253202.

[7] C. M. Clausen, M. L. S. Nielsen, J. K. Pedersen, J. Rossmeisl, *High Entropy Alloys & Materials* **2023**, *1*, 120–133.

[8] M. L. S. Nielsen, J. K. Pedersen, M. F. Nygaard, M. K. Plenge, H. H. Kristoffersen, J. Rossmeisl, *Faraday Discussions* **2026**.

[9] M. K. Plenge, J. K. Pedersen, L. A. Cipriano, J. Rossmeisl, *High Entropy Alloys & Materials* **2025**, *3*, 165–177.

[10] I. E. L. Stephens, A. S. Bondarenko, U. Grønbjerg, J. Rossmeisl, I. Chorkendorff, *Energy & Environmental Science* **2012**, *5*, 6744.

[11] T. Prohaska, J. Irrgeher, J. Benefield, J. K. Böhlke, L. A. Chesson, T. B. Coplen, T. Ding, P. J. H. Dunn, M. Gröning, N. E. Holden, et al., *Pure and Applied Chemistry* **2022**, *94*, 573–600.

[12] K. Yang, M. Emmerich, A. Deutz, T. Bäck, *Journal of Global Optimization* **2019**, *75*, 3.

[13] M. Balandat, B. Karrer, D. Jiang, S. Daulton, B. Letham, A. G. Wilson, E. Bakshy, *Advances in neural information processing systems* **2020**, *33*, 21524.

[14] S. Ament, S. Daulton, D. Eriksson, M. Balandat, E. Bakshy, *Advances in Neural Information Processing Systems* **2023**, *36*, 20577.

[15] S. Daulton, M. Balandat, E. Bakshy, *Advances in neural information processing systems* **2021**, *34*, 2187.

[16] J. K. Pedersen, C. M. Clausen, O. A. Krysiak, B. Xiao, T. A. A. Batchelor, T. Löffler, V. A. Mints, L. Banko, M. Arenz, A. Savan, et al., *Angewandte Chemie International Edition* **2021**, *60*, 24144–24152.
